# Application of confocal surface wave microscope to self-calibrated attenuation coefficient measurement by Goos-Hänchen phase shift modulation

**DOI:** 10.1038/s41598-018-26424-2

**Published:** 2018-06-04

**Authors:** Suejit Pechprasarn, Terry W. K. Chow, Michael G. Somekh

**Affiliations:** 1 Department of Electronic and Information Engineering, The Hong Kong Polytechnic University, Hung Hom, Hong Kong SAR China; 2 Faculty of Biomedical Engineering, Rangsit University, Pathum Thani, 12000 Thailand; 3 Nanophotonics Research Center, Shenzhen University, Shenzhen, China; 4 Faculty of Engineering, University of Nottingham, Nottingham, NG7 2RD UK

## Abstract

In this paper, we present a direct method to measure surface wave attenuation arising from both ohmic and coupling losses using our recently developed phase spatial light modulator (phase-SLM) based confocal surface plasmon microscope. The measurement is carried out in the far-field using a phase-SLM to impose an artificial surface wave phase profile in the back focal plane (BFP) of a microscope objective. In other words, we effectively provide an artificially engineered backward surface wave by modulating the Goos Hänchen (GH) phase shift of the surface wave. Such waves with opposing phase and group velocities are well known in acoustics and electromagnetic metamaterials but usually require structured or layered surfaces, here the effective wave is produced externally in the microscope illumination path. Key features of the technique developed here are that it (i) is self-calibrating and (ii) can distinguish between attenuation arising from ohmic loss (*k*″_*Ω*_) and coupling (reradiation) loss (*k*″_*c*_). This latter feature has not been achieved with existing methods. In addition to providing a unique measurement the measurement occurs of over a localized region of a few microns. The results were then validated against the surface plasmons (SP) dip measurement in the BFP and a theoretical model based on a simplified Green’s function.

## Introduction

Optical surface waves occur when light is guided by a surface; these waves then propagate parallel to the surface with its energy confined close to that surface. Examples of this are, of course, surface plasmons (SP), surface waves guided by multilayered waveguide structures and some gratings. In this paper, we propose an approach to measure attenuation coefficients of surface waves. Recently, there has been great interest in optical surface wave structures, such as, surface plasmon waveguide structures^1,2^ and Dykonov surface wave structures^3^ for biosensing, optical computing and nonlinear optics enhancement. The attenuation coefficients play a crucial role in designing and characterizing such optical devices, for example, in waveguides^4^ and surface plasmon sensors and microscopes^5,6^. Here we show that a confocal microscope configuration integrated with a phase-SLM can provide an accurate measurement of attenuation coefficients and quantitatively separate each loss mechanism. To the best of our knowledge, an attempt to separate and quantify the ohmic and the coupling loss mechanisms by direct measurement has never been reported before either in near or far field measurement and certainly not within a microscope structure. Moreover, the measurement approach presented here shows how the SLM provides a means to perform an internal calibration of the required microscope parameters that would otherwise be extremely difficult to evaluate accurately. Although this paper concentrates on attenuation measurement the idea of using a self-calibrated instrument should have applications in the measurement of other parameters on a local scale. For instance, Jayasurya *et al*.^7^ compare the mean displacement of *p-* and *s-* polarized beams to measure the Goos-Hänchen shift, the use of the reference beam allows measurements of the shift with precision of about 100 nm. In the present paper the reference beam provides an internal calibration that allows the different mechanisms of SP attenuation to be separated. Moreover, measurement through a microscope objective allows SP to be measured independently of a directly reflected beam, whereas the Goos-Hänchen effect with wide beams involve overlapping and interfering contributions from the directly reflected and SP contributions.

In this paper, the SP has been chosen as an example to demonstrate the method. The SP wave phenomena has been widely studied. It is very well established that the SP dissipates its energy through two major loss mechanisms^8^ (i) coupling loss due to coupling and reradiation (*k*″_*c*_) and (ii) ohmic loss due to the SP propagation on a lossy material (*k*″_*Ω*_). Although we focus on the SP, the method is applicable to a wide range of other surfaces as we discuss later in the paper.

There are a number of methods to measure the attenuation coefficient including a near field measurement using a scanning near field optical microscope (SNOM)^9^, and far field methods, such as, photoemission microscopy imaging^10^, fluorescent imaging^11^, pump-probe experiment^12^ and confocal microscopy^13^. The near field approach requires a scanning tip, which perturbs the measurement and obscures the accessibility to the sample. Although the far field methods measure the SP propagation profile directly, they do have some limitations. For photoemission microscopy imaging, a high power pulsed laser is required for nonlinear photoemission of metal film; for fluorescent imaging, the fluorescent dye will, of course, alter the excitation condition of the SP and also suffer from differential photobleaching along the propagation path. For the pump-probe experiment, the accuracy of the measurement often suffers from a poor signal to noise ratio. Recently, we have demonstrated that a modified confocal microscope^13^ can be employed to measure the attenuation of the SP. It is important to note that, so far, none of these direct methods including our own confocal microscope could separate the two loss mechanisms. Here, we propose, for the first time, a phase modulation methodology to quantitatively separate the loss mechanisms. The method presented can be regarded as a bridge between near field methods and low spatial resolution far field measurements.

Another approach involves indirect evaluation of the parameters by using a model to fit to the loss parameters. The indirect approach essentially relates the reflection coefficient with the attenuation coefficient. There are a number of publications developing theoretical models to explain the relationship between the amplitude and phase of the SP reflection spectrum and its attenuation including Goos-Hänchen phase shift model^14^, pole and zeros model^15^, Poynting vector^16^, multilayered structure model^17,18^ and a simplified Green’s function^19,20^. All these methods involve fitting to a model which require detailed knowledge of the structure, clearly our direct measurement simply measures the generated waves without any presumptions about the structure.

## Materials and Methods

### Simplified Green’s function

In this paper, we employ the simplified Green’s function described in ref.^19^ to explain key features of the Goos- Hänchen phase shift of the surface wave and demonstrate some issues in the amplitude measurement without going through complicated equations. It must be pointed out, however, that this model is used for explanation and validation. It is not a necessary part of the measurement process which does not rely on any model. For the simplified Green’s function, the SP reflection dip as a function of incident *k-*vector along × direction *R*_*p*_*(k*_*x*_*)* can be expressed as an interference sum of direct reflection, *R*_*d*_*(k*_*x*_*)*, and the SP, *R*_*sp*_*(k*_*x*_*)*, which is given by (see Supplementary materials S3):

1 $${R}_{p}({k}_{x})={R}_{d}({k}_{x})+{R}_{sp}({k}_{x})=-\,1+2{k}_{c}^{^{\prime\prime} }{r}_{sp}({k}_{x},{k}_{sp})$$

2 $${r}_{sp}({k}_{x},{k}_{sp})=\frac{i}{{k}_{sp}-{k}_{x}}+\frac{i}{{k}_{sp}+{k}_{x}}$$

where *R*_*d*_*(k*_*x*_*)* is the direct reflection from plasmonic metal assumed to be −1 for simplicity. The term *R*_*sp*_*(k*_*x*_*)* is the contribution to the reflection coefficient due to the excitation of the SP reflection on the surface of the metal. *k*_*sp*_ is the complex SP *k-*vector *k*_*sp*_ = *k*′_*sp*_ + *ik*″_*sp*_. The strongest SP excitation occurs when *k*_*x*_ matches the real part of *k*_*sp*_. The term *k′*_*sp*_ is the SP *k-*vector and *k*″_*sp*_ gives the total attenuation of the SP consisting of two loss mechanisms *k*″_*sp*_ = *k*″_*c*_ + *k*″_*Ω*_.

3 $$SP(x)=|A\gamma {e}^{ix{k}_{sp}}|=|A\gamma {e}^{ix{k}_{sp}^{^{\prime} }}{e}^{-x{k}_{sp}^{^{\prime\prime} }}|$$

Where *SP* is the magnitude of the SP wave as a function of propagation distance *x*. *A* is related to the value of *k*″_*c*_ and $\gamma$ is an instrument dependent parameter that depends on factors, such as, the optical power and the responsivity of the detection process. *k*″_*Ω*_ and *k*″_*c*_ are the ohmic attenuation coefficient and the coupling attenuation coefficient respectively. The terms ${e}^{ix{k}_{sp}^{^{\prime} }}$ and ${e}^{-x{k}_{sp}^{^{\prime\prime} }}$ are the SP propagation phase term and the amplitude damping term respectively. In a full wave analysis, the two coupling mechanisms for loss cannot be readily separated. Some estimate, however, can be made by performing the reflectivity calculation with the imaginary part of the metal permittivity set to zero. In this case, there is only coupling loss, although the values obtained are not exactly representative of the real metal because the change in the dielectric properties will (slightly) change the coupling.

**Figure 1:**
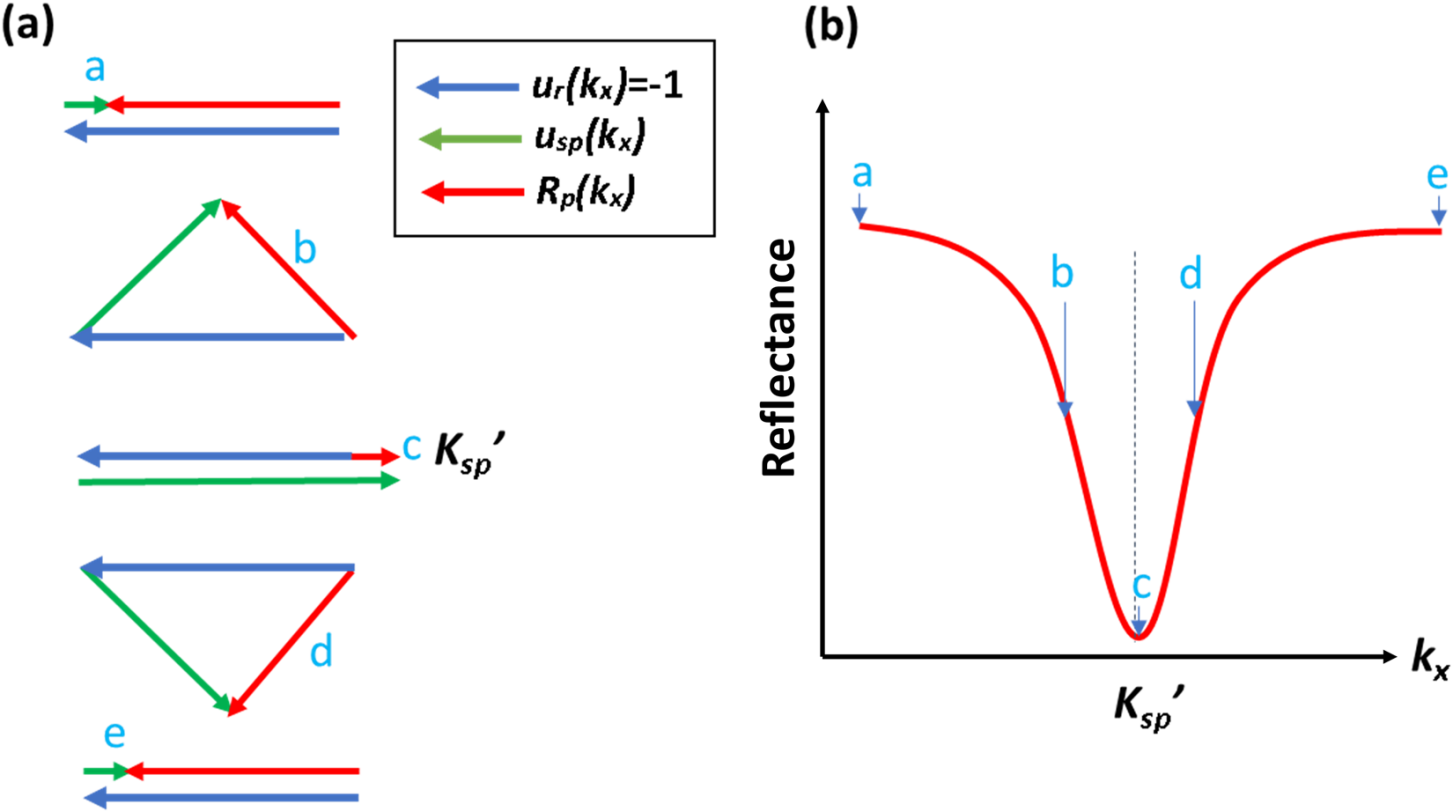
Shows conceptual diagram of the simplified Green’s function model. (**a**) *R*_*p*_*(k*_*x*_) vector sum shown in red of the direct reflection term, *u*_*r*_*(k*_*x*_) in blue and the SP term, *u*_*sp*_*(k*_*x*_) in green for different incident angles and (**b**) resultant vector *R*_*p*_*(k*_*x*_) as a function of incident *k*-vector *k*_*x*_

The SP dip calculated using the simplified Green’s function *R*_*p*_*(k*_*x*_) can be represented as a sum of 2 vectors the *u*_*r*_*(k*_*x*_) (−1 depicted in blue in Fig. 1a) and the *u*_*sp*_*(k*_*x*_) (the SP depicted in green with π rad phase shift in Fig. 1a, where the resultant vector (reflection coefficient) is depicted in red in Fig. 1a,b.

**Figure 2:**
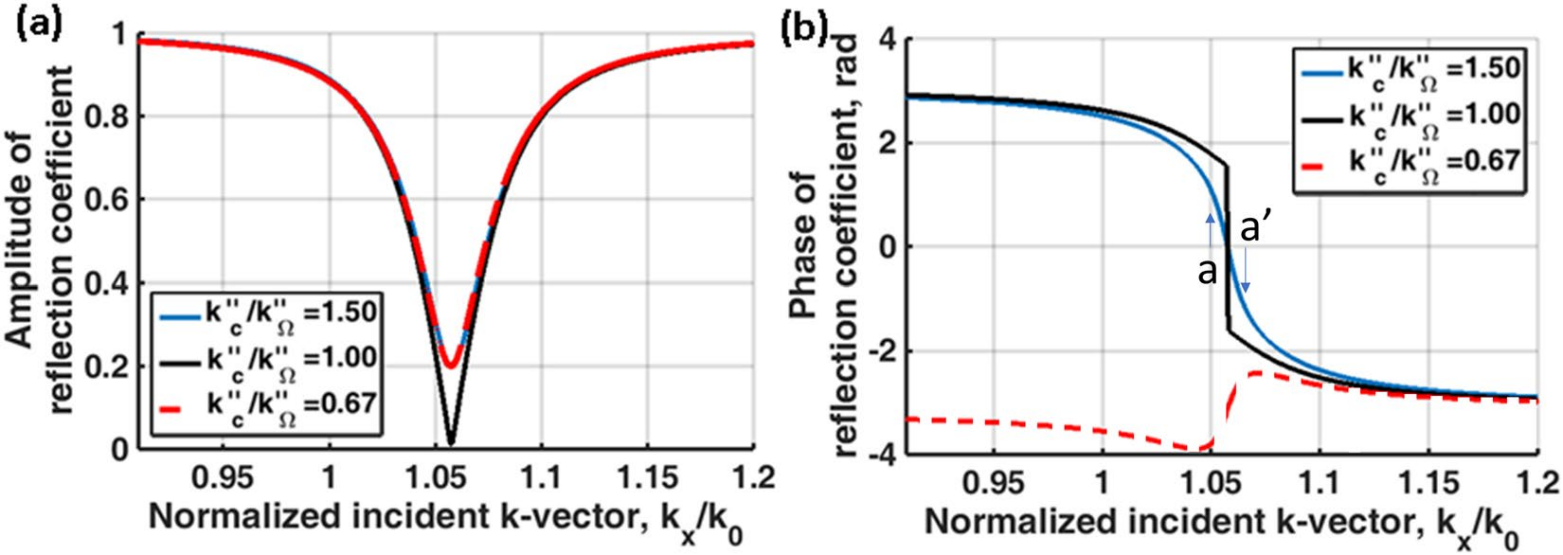
Shows (**a**) amplitude of reflection coefficient and (**b**) phase of reflection coefficient when *k*″_*c*_*/k*″_*Ω*_ = *1*.*50* (blue curve), *k*″_*c*_*/k*″_*Ω*_ = *1*.*00* (black curve) and *k*″_*c*_*/k*″_*Ω*_ = *0*.*67* (red curve).

Figure 2 shows amplitude and phase of reflection spectra for 3 cases; where *k*″_*sp*_ = 0.*018k*′_*sp*_ with different ratios of coupling to ohmic loss (i) *k*″_*c*_/*k*″_*Ω*_ = 1.50, (ii) *k*″_*c*_/*k*″_*Ω*_ = 1.00 and (iii) *k*″_*c*_/*k*″_*Ω*_ = 0.67. Of course, the absolute values of *k*″_*c*_ and *k*″_*Ω*_ are also important with large values leading to a less sharp transition. The amplitudes of reflection spectra of the cases (i) and (iii) have a very similar amplitude profile leading to an ambiguity in amplitude detection, whereas the phase responses are different. In other words, they have different Goos-Hänchen phase shifts^14^ (proportional to −*dφ/dk*_*x*_). The phase information does play a crucial role in separating the *k*″_*c*_ and *k*″_*Ω*_, we will explain later how the confocal system allows the phase information in the BFP to be revealed.

The form of the reflection coefficient in Fig. 2 leads to the Goos-Hänchen shift. As explained in Yallapragada *et al*.^21^ the phase variation leads to the spatial GH shift where the energy is displaced along the surface. A gradient in the magnitude of the reflectivity leads to a change in the mean angle of reflection. Essentially, this can be thought of as arising from different incident angles suffering different reflection coefficients, so that the weighted average angle of reflection is changed. In our SP experiments, both of these effects will occur, however, it is primarily the spatial shift and the corresponding axial displacement that is of interest. Here we are concerned with the change of surface wave intensity as it propagates and the small change in angle makes a negligible change to the propagation distance.

The GH shift is considered in many papers including an exhaustive review by Bliokh *et al*.^22^. In the simplest form GH shift involves an oblique paraxial beam whose spatial frequencies interact with an approximately linear variation in phase shift as depicted by points a and a´ in the blue curve of Fig. 2b. If the reflected intensity is constant, the phase variation results in a simple displacement of the beam without distortion. Variations in the amplitude and the linearity of the phase clearly introduce considerable distortions of the beam. In our system, the underlying physics fits entirely within the framework of the GH effect, however, the complex beam profile with many spatial frequencies and phase variation of the input field due to defocus means the GH phenomenon manifests itself in a somewhat different way from the conventional experiments. In essence, the region where the phase variation of the input beam matches the *k*-vector of the input beam generates a wave that propagates along the surface. Like the more usual manifestation of the spatial GH shift this represents a movement of the centroid of the reflected beam relative to the incident beam but in this case the profile of the surface wave can be seen explicitly. This is analyzed in more detail in the Supplementary materials (S5).

### A modified confocal microscope and attenuation measurements

**Figure 3:**
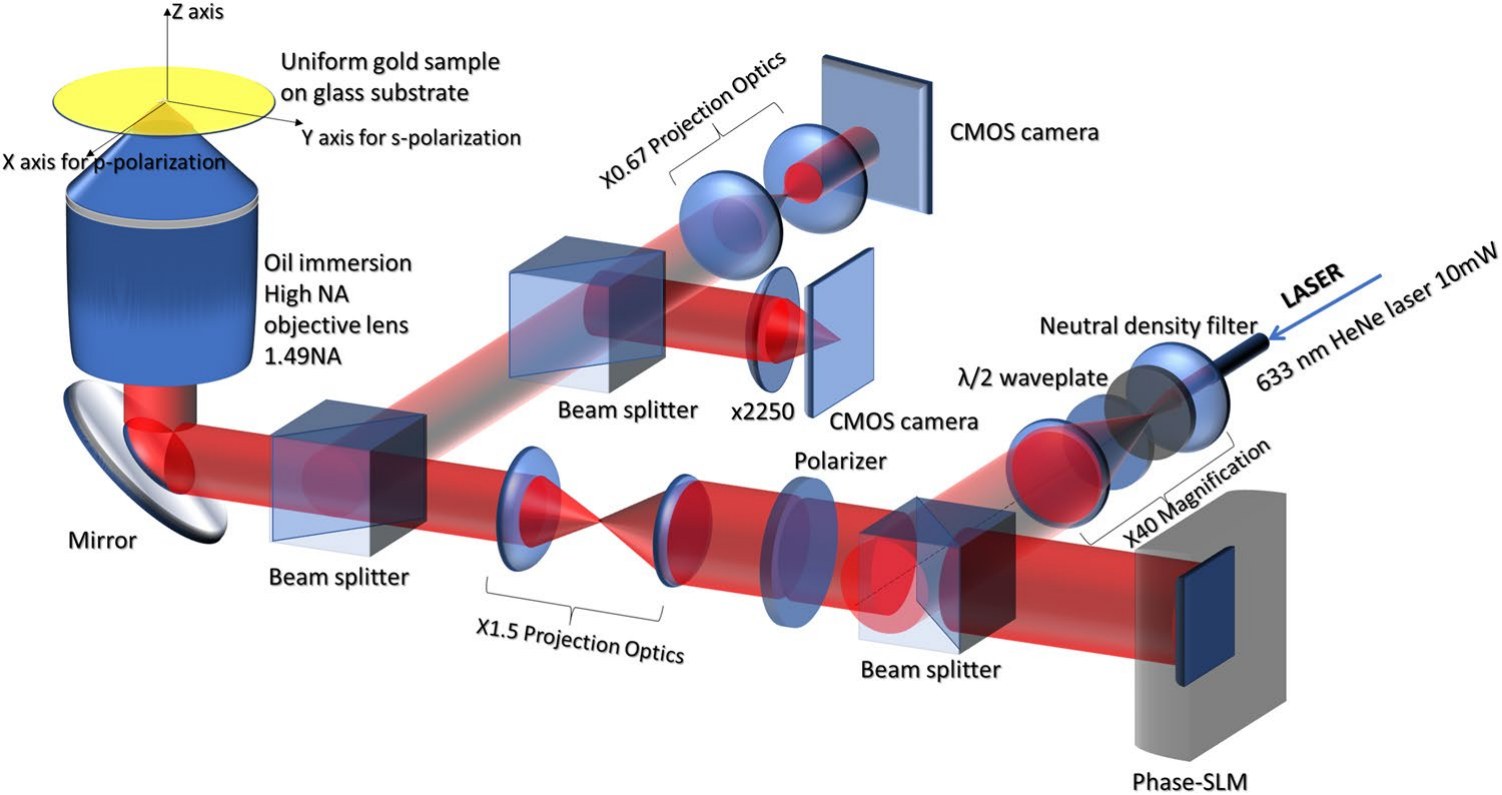
Shows a system diagram of confocal surface plasmon microscope showing all optical components in the system, xyz axes and polarizations corresponding to each axis.

**Figure 4:**
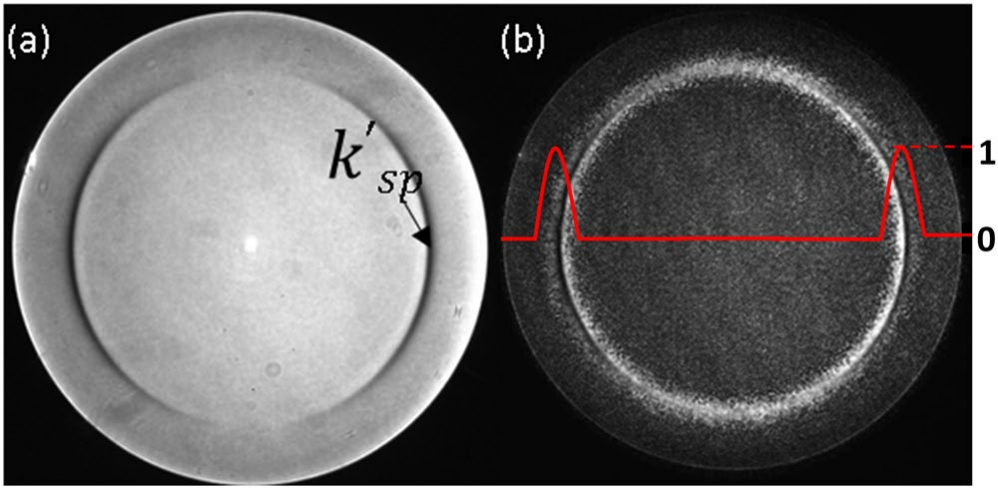
Shows (**a**) experimental BFP image for 46 nm thick Au sample, (**b**) experimental BFP image with amplitude pupil function modulation using phase-antiphase pairs pattern on the phase-SLM; the amplitude pupil function is shown in red. The outer edge of the BFP corresponds to a NA of 1.49 in agreement with the manufacturers specification.

Recently, we have demonstrated the use of a modified confocal SP microscope as shown in Fig. 3 to measure the *k*′_*sp*_ and the total attenuation coefficient of the SP^13^ without separating the different loss mechanisms. In the current system setup, we replaced the confocal pinhole and the photodetector with a CMOS camera allowing us to electronically control the pinhole size by integrating only the light intensity of the pixels within a required pinhole size. A phase-SLM in a conjugate plane to the BFP has been incorporated into the system to enable (i) defocusing without mechanical scanning by providing a defocused phase pattern on the phase-SLM^23^ and (ii) provide an amplitude pupil function using phase-antiphase pairs pattern on the phase-SLM^13^ as shown in Fig. 4b. The light source used in the system is a linear polarized laser at 633 nm wavelength (*λ*_*0*_); where the corresponding SP wavelength (*λ*_*sp*_) is 599 nm (633 nm/1.057). The system employs a 1.49NA oil immersion objective lens with oil immersion refractive index (*n*) 1.52, which provides sufficient *k-*vector to excite the SP as the SP dip can be clearly observed in the BFP image as shown in Fig. 4a, where the SP dip is labelled *k*′_*sp*_. Further details of the optical configuration can be found in the Supplementary material (S1). The hardware configuration used in the present paper is similar to previous work^24^, however, the measurement approach is radically different as we show here how the microscope can be used to perform its own internal calibration.

**Figure 5:**
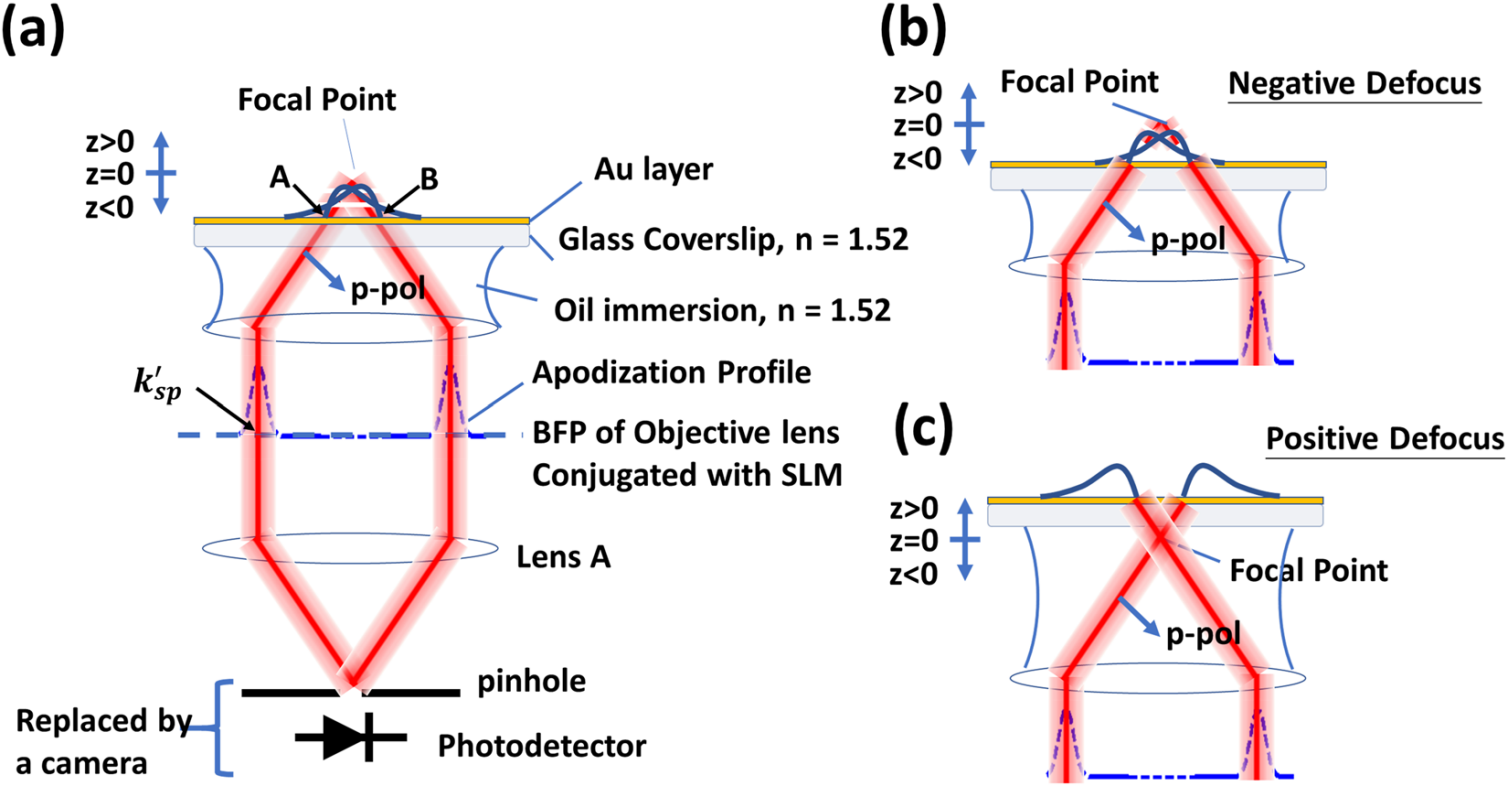
Shows (**a**) a conceptual schematic diagram of the confocal SP microscope, (**b**) negative defocus where the SP propagates towards the center of the optical axis and (**c**) positive defocus where the SP propagates away from the optical axis

For the defocused illumination and *p-*polarized light, the *k-*vector position *k*′_*sp*_ (=2*πnsinθ*_*p*_/*λ*_0_) shown in conceptual diagram in Fig. 5a, the SP is excited at ‘A’ and propagates to ‘B’ and beyond, but only the SP that appears to come from the focus will pass through the confocal pinhole. This gives a well-defined propagation path of the SP allowing us to have a localized measurement over a small region^6^. The attenuation measurement is carried out with the central part of the BFP blocked by providing an amplitude pupil function modulation as shown in Fig. 4b. The confocal signal is then measured as the sample is defocused towards the objective lens (negative defocus as shown in Fig. 5b), the so called, *V(z*). The full derivation of the *V(z*) response is given in the Supplementary Materials (S4) where we point out that the overall response is dependent on the product of the lens pupil function, the phase factor arising from defocus and the reflection coefficient, identical responses can be therefore obtained for similar products of these parameters regardless of the value of the individual terms. Since the SLM allows us to control the pupil function we can use the SLM to replicate the effect of different defocuses and sample response functions.

For the positive defocus, the SP propagates away from the center of the optical axis and will miss the pinhole as shown in Fig. 5c. The amplitude pupil function windows out the angular frequencies not contributing to the SP signal and also enables us to measure samples with multi-modes by tuning the center of the annulus window. The window has been designed so that it is sufficiently wide to cover the phase transition of the SP, however, it must not be too narrow. If the pupil function is too narrow, the direct reflection will not be blocked by the pinhole thus affecting the accuracy of the SP measurement. The detailed explanation of this effect can be found in Supplementary material section (S4).

**Figure 6:**
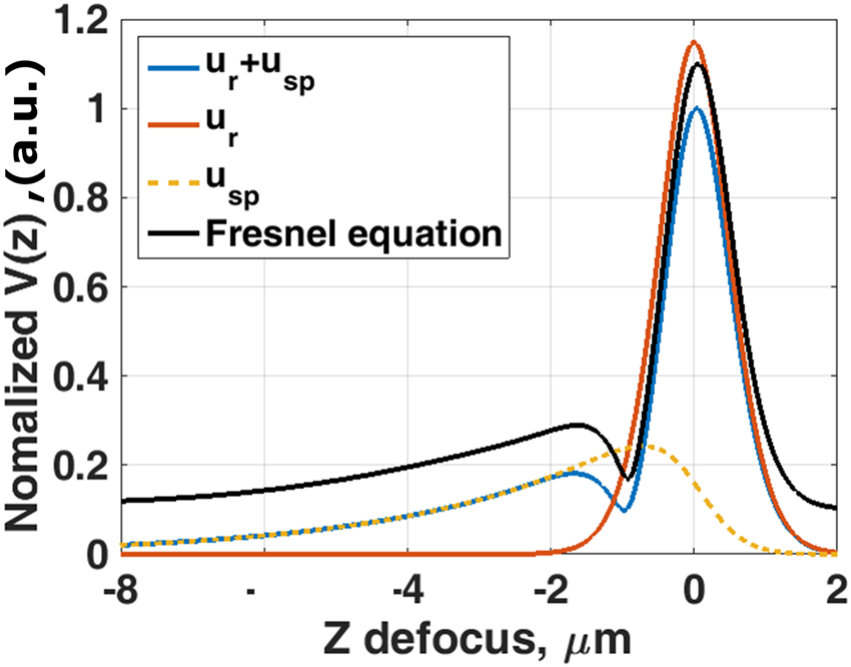
Shows simulated normalized *V(z*) for the BFP calculated using Eq. (1) with *k*″_*sp*_ = *0*.*018k*′_*sp*_, *k*″_*c*_/*k*″_*Ω*_ = 2.50, *nsinθ*_*p*_ = 1.0570, where *n* is the refractive index of immersion oil of 1.52, and the amplitude pupil function shown in Fig. 4b. The dashed blue curve is the *V(z*) curve calculated by for the simplified model BFP using Eqs (1) and (2); the red curve is the *V(z*) calculated from the simplified model BFP with the *u*_*sp*_*(k*_*x*_) = 0; the dashed yellow curve is the *V(z*) calculated from the simplified model BFP with the *u*_*r*_*(k*_*x*_) = 0 and the solid black curve is the *V(z*) calculated from the BFP using Fresnel equation for 40 nm of uniform gold with refractive index of 0.1961 + 3.2558i^44^ for 633 nm incident wavelength. The *V(z*) calculated from the BFP using the Fresnel equations (the black curve) was offset by 0.1 for clarity.

Figure 6 shows the *V(z*) confocal responses of a BFP calculated using Eq. (1), where *k*″_*sp*_ = *0*.*018k*′_*sp*_, *k*″_*c*_/*k*″_*Ω*_ = 1.50, *nsinθ*_*p*_ = 1.0570 corresponding to each of the terms *u*_*r*_(*k*_*x*_*)* (red curve), *u*_*sp*_*(k*_*x*_*)* (dashed yellow curve) and *R*_*p*_*(k*_*x*_*)* (dashed blue curve); where *θ*_*p*_ is the surface plasmon angle. It is particularly illustrative to use this form of reflectance function since the surface wave and non-surface wave contribution are written explicitly and separately, allowing the physical origin of the response to be measured directly. When the microscope is defocused by more than −3 µm from the focal point towards the objective lens; the direct reflection misses the pinhole allowing us to measure the SP wave alone. When the two contributions cannot be mathematically separated explicitly, for the reflection arising from the Fresnel equation (the black curve), we can be confident that when the sample is defocused beyond −3 μm towards the objective that the dominant contribution comes from the excited surface waves.

**Figure 7:**
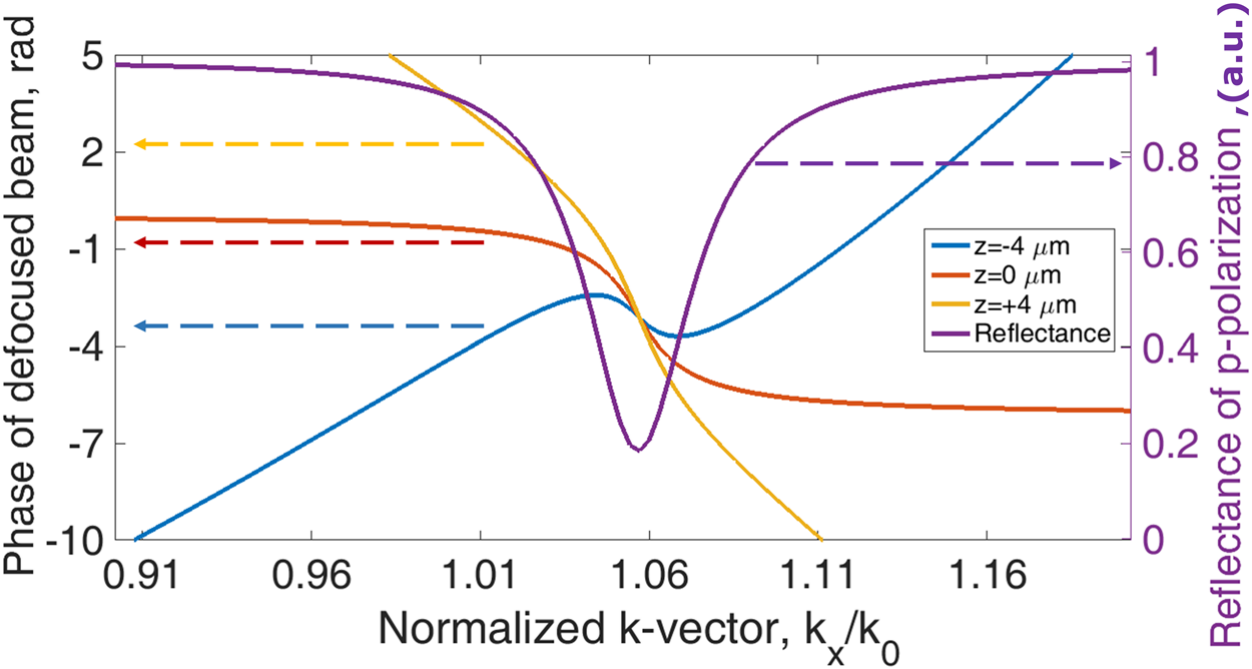
Shows the phase of the reflected defocused beam at z = −4 µm (in blue), 0 µm (in red) and +4 µm (in yellow). The defocus modulates the phase change due to SP excitation so that the phase gradient is reduced, thus leading to a larger contribution to the output.

Having mentioned that the phase information around the SP dip is crucial to distinguish the loss mechanisms as explained in the GH section. The confocal system does not recover the phase information in the BFP directly, but it does, in fact, modulate the phase around the SP by the defocusing phase transfer function, *ϕ(θ)*, of *2kzcosθ*^23^, where *k* is the *k-*vector in the coupling medium, *z* is the defocus distance and *θ* is the incident angle in the BFP. To illustrate this point, let us take one of the phase profiles in Fig. 2b in this case the case *k*″_*c*_*/k*″_*Ω*_ = 1.50 and modulate this phase profile by the defocusing phase transfer function of *2kzcosθ* at (i) negative defocus at z = −4 µm, (ii) in focus z = 0 µm and (iii) positive defocus at z = 4 µm as shown in Fig. 7. It can be clearly seen that the defocusing phase transfer function at z = −4 µm flattens the phase around the SP phase transition allowing the SP effect to be emphasized and measured using the confocal system. In mathematical terms, this means that the regions where, $\tfrac{d{\varphi }(\theta )}{d{k}_{x}}\approx 0$ make the largest contribution by the stationary phase principle^25^ (Supplementary material S5).

### Total attenuation measurement under the confocal microscope configuration

In view of the considerations above for sufficient defocus beyond −3 µm shown in Fig. 6, the SP signal detected can be expressed as:

4 $$SP(z)=A\gamma {e}^{-z({k}_{cz}^{^{\prime\prime} }+{k}_{{\rm{\Omega }}z}^{^{\prime\prime} })}=2{k}_{cz}^{^{\prime\prime} }\gamma {e}^{-z({k}_{cz}^{^{\prime\prime} }+{k}_{{\rm{\Omega }}z}^{^{\prime\prime} })}$$

Where *SP(z)* is the magnitude of SP wave as a function of defocus *z*. *A* is related to the value of *k*″_*cz*_, where *A* can be written as *2k*″_*cz*_; *k*″_*Ωz*_ and *k*″_*cz*_ are the ohmic attenuation coefficient and the coupling attenuation coefficient as a function of *z* defocus respectively. The terms *k*″_*Ωz*_ and *k*″_*cz*_ can be converted to the attenuation coefficients with the direction parallel to the surface of metal *k*″_*Ωz*_ and *k*″_*c*_ by dividing the terms *k*″_*Ωz*_ and *k*″_*cz*_ by 2tan*θ*_*p*_. $\gamma$ is an instrument dependent parameter which must be known (or eliminated) to separate the two attenuation coefficients from the measured decay curve. The parameter *γ* depends on factors, such as, the optical power, the pupil function, the responsivity of the detection process and the pinhole size. In principle, this parameter could be measured directly, however, the cumulative errors from such a process would render the measurement unusable. The key in this paper is to generate an artificial surface wave with known parameters. Since this wave is generated in the same microscope it will have the same *γ*, this provides a self-calibrated internal measurement of this parameter. This process is discussed in detail later in the paper where the separation of the two attenuation parameters is discussed.

By taking natural logs of both sides of equation (4):

5 $$\mathrm{ln}(SP(z))=\,\mathrm{ln}\,A+\,\mathrm{ln}\,\gamma -z({k}_{cz}^{\text{'}\text{'}}+{k}_{{\rm{\Omega }}z}^{\text{'}\text{'}})=\,\mathrm{ln}(2{k}_{cz}^{\text{'}\text{'}})+\,\mathrm{ln}\,\gamma -z({k}_{cz}^{\text{'}\text{'}}+{k}_{{\rm{\Omega }}z}^{\text{'}\text{'}})$$

We can therefore see that the gradient gives total attenuation.

It is important to note that the *V(z)* confocal measurement did not require the value of refractive index of gold to work out the attenuation coefficient, in other words, the *V(z)* confocal measurement is a model-free method. The attenuation is observed at negative defocus because when the phase and group velocities are in the same direction the surface waves are only detected at negative defocus. The corollary of the effect when the phase and group velocities oppose each other as used later in this paper; the surface waves are detected with positive defocus.

### Confocal surface plasmon microscope as a tool to determine the complex permittivity of gold

**Figure 8:**
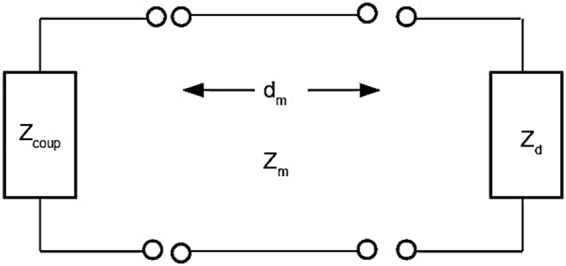
Shows equivalent circuit for the SP resonance.

In this section, we will demonstrate that the *V(z)* confocal measurement can be employed as a tool to measure the complex permittivity of gold (*ε*_*m*_). The idea behind this is to solve for real and imaginary parts of the wavenumber of the SP as function of *ε*_*m*_. We do this using a transmission line equivalent circuit and solving for the transverse resonance conditions^26^ of the mode, where the SP samples were treated as an equivalent circuit consisting of two impedances and a transmission line representing the gold layer as shown in Fig. 8.

The relative impedances are expressed as:

6 $${Z}_{m}=\,\cos \,{\theta }_{m}/\sqrt{{\varepsilon }_{m}}$$

7 $${Z}_{d}=\,\cos \,{\theta }_{d}/\sqrt{{\varepsilon }_{d}}$$

8 $${Z}_{coup}=\,\cos \,{\theta }_{coup}/\sqrt{{\varepsilon }_{coup}}$$

9 $${Z}_{end}={Z}_{m}\frac{{Z}_{coup}+{Z}_{m}\,\tanh (i{k}_{zm}{d}_{m})}{{Z}_{m}+{Z}_{coup}\,\tanh (i{k}_{zm}{d}_{m})}$$

where *Z*_*end*_ is the impedance of the coupling medium projected through the metal layer, *ε*_*d*_, *ε*_*m*_ and *ε*_*coup*_ are the permittivity of the dielectric on the top of metal (air in this case *ε*_*d*_ = 1), the metal and the dielectric coupling media respectively. *Z*_*m*_, *Z*_*d*_ and *Z*_*coup*_ are the impedances of the metal layer, the dielectric layer on the top of metal and the coupling layer respectively. *cosθ*_*m*_, *cosθ*_*d*_ and *cosθ*_*coup*_ are cosine values of the complex angles of refraction for the metal layer, the dielectric layer on the top of metal and the coupling layer respectively. *k*_*zm*_ is the *k-*vector along z direction of the metal layer, which is given by *k*_*zm*_ = *k*_*m*_*cosθ*_*m*_. *k*_*m*_ is the *k-*vector in the metal layer, which is given by ${k}_{m}=2\pi \sqrt{{\varepsilon }_{m}}/{\lambda }_{0}$ where *λ*_0_ is the free space wavelength of the incident beam. *d*_*m*_ is the thickness of the metal film. For a given set of *ε*_*d*_, *ε*_*m*_, *ε*_*coup*_, *λ*_0_ and *d*_*m*_, we can solve for complex *sinθ*_*d*_ that satisfies the transverse resonance condition *Z*_*d*_ + *Z*_*end*_ = 0, where the impedances each side of the reference plane are propagated in different direction. This enables us to determine complex *k-*vector of the SP *k*_*sp*_ = *k*_*d*_*sinθ*_*d*_, where *k*_*d*_ is given by ${k}_{d}=2\pi \sqrt{{\varepsilon }_{d}}/{\lambda }_{0}$.

For each known thickness, we then varied both real part and imaginary part of *ε*_*m*_ ranging from −10 to −8 and 0i to 2i for real part and imaginary part respectively calculating contour maps of complex *k*_*sp*_ solutions. In this part, the complex *k*_*sp*_ solutions were separated into two contours (1) *nsinθ*_*d*_ (SP dip position in the BFP) and (2) *1/k*″_*sp*_. The intersection between the two conditions (a) and (b) gives the complex permittivity of the gold as demonstrated in Results section.

### Separating SP loss mechanisms using Goos-Hänchen phase shift engineering using a phase spatial light modulator

Up to this point, we have explained how the confocal system can perform rapid measurements of attenuation that complement other techniques by performing the measurement in a microscopic context in a small localized region. In this section, we will show that the flexibility conferred by the SLM in the confocal microscope allows us to separate the two major contributions to attenuation.

From Eq. (5), the slope and the intercept of the linear fit through the natural log scale tell us about *k*″_*Ωz*_ + *k*″_*cz*_ and ln(2k″_*cz*_) + lnγ respectively. To determine *k*″_*cz*_, we need the intercept but we can only get the intercept if we know *γ*. Assessing *γ* from instrumental conditions is highly cumbersome and fraught with errors, as discussed above, however, if we can generate a “wave’’ with a known *A* within the same microscope we can then measure *γ* in a self-consistent way. This will be demonstrated through Goos-Hänchen phase shift engineering using phase spatial light modulator.

The additional independent measurement utilizes the linear polarization in the system, where the *p-*polarization excites the SP and the *s-*polarization does not excite the SP as can be seen along the horizontal and vertical directions in Fig. 4a,b. We can use the SLM to impose a phase profile on the incident beam that effectively generates an artificial surface wave on the sample surface. By imposing a phase profile given by:

10 $${\varphi }({k}_{x})=2\,{\tan }^{-1}(a({k}_{x}+{k}_{p}))+\pi $$

where *ϕ(k*_*x*_) is the artificial surface wave phase gradient superimposed on the phase-SLM. *k*_*p*_ and *a* are the variables that determines the mean position and the gradient of the phase transition respectively. There is no essential requirement to use the arctan phase function, however, the arctan phase function gives a good representation of surface wave phase profile. Not only that, the tan^−1^ phase function can also be realized by metamaterial structures, for example, grating structure reported by Vasića and Gajić^27^.

**Figure 9:**
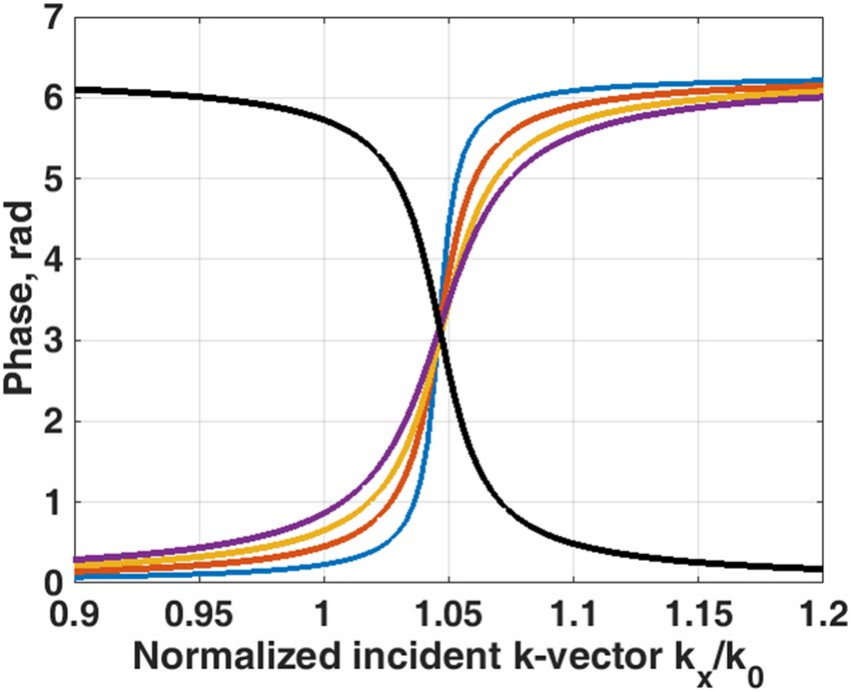
Shows the SPs phase in black curve and the artificial surface wave phases calculated by Eq. (10) with the following parameters *(a*, *k*_*p*_) = *(19e3*, *0*.*0104*) for blue curve, *(a*, *k*_*p*_*)* = *(9*.*5e3*, *0*.*0104)* for red curve, *(a*, *k*_*p*_*)* = *(6*.*3e3*, *0*.*0104)* for yellow curve and *(a*, *k*_*p*_*)* = *(4*.*7e3*, *0*.*0104)* for purple curve.

**Figure 10:**
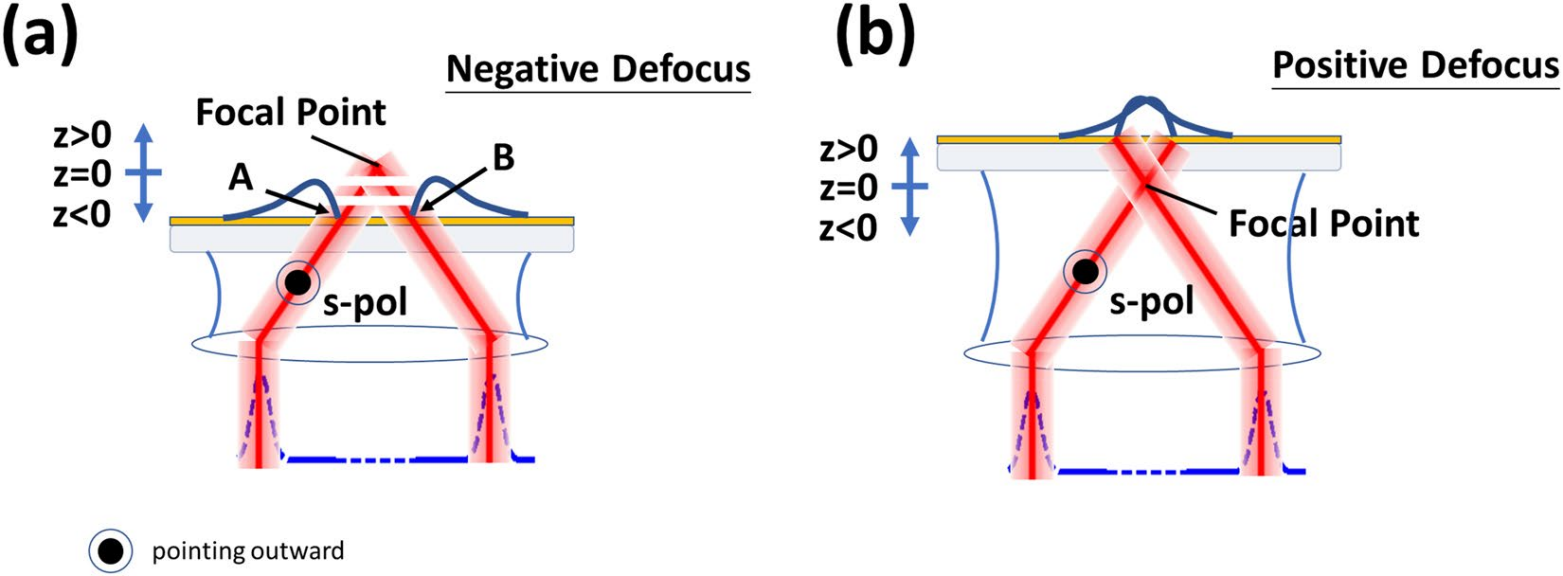
Shows (**a**) negative defocus where the *s-*polarization artificial surface wave propagates away from the optical axis and (**b**) positive defocus where the s-polarization artificial surface wave propagates towards the optical axis.

For the *s-*polarization, the wave vector (*k*′_*sp*_) at the phase transition position was the same as the SP, but the phase transition was, however, opposite to the SP as shown in Fig. 9. In other words, the Goos-Hänchen phase shift −*dφ*/*dk*_*y*_ (*y* subscript since the *s-*polarization is orthogonal to the *p-*polarization) was opposite to the SP leading to an opposite rotation of the surface wave phasor (opposite rotation to Fig. 1a) so that this this artificial surface wave effectively propagates backwards as shown in Fig. 10. In the confocal system, this wave can only be collected by the objective lens with positive defocus as shown by the diagram in Fig. 10b. This backward artificial wave can be described by ${k}_{ASP}={k^{\prime} }_{sp}-i{k}_{ASP}^{^{\prime\prime} }$, where *k*_*ASP*_ is the artificial surface wave *k-*vector and *k*″_*ASP*_ is the attenuation coefficient of the artificial surface wave. Consequently, it excites an *s*-polarized artificial surface wave. The backward surface wave with negative group velocity has been realized in acoustics with a backward propagating Rayleigh wave^28^ and recently realized in optics using metamaterial^29^. Here we have demonstrated a way to mimic this backward surface wave effect using a spatial light modulator with no need for a sophisticated metamaterial structure fabrication.

11 $$ASP(y)=|\gamma 2{k}_{c,ASP}^{^{\prime\prime} }{e}^{iy{k}_{ASP}}|=|\gamma 2{k}_{c,ASP}^{^{\prime\prime} }{e}^{iy{k}_{sp}^{^{\prime} }}{e}^{y{k}_{sp}^{^{\prime\prime} }}|$$

where *ASP* is the magnitude of artificial surface wave. The term ${e}^{iy{k}_{sp}^{^{\prime} }}$ is the phase term and ${e}^{y{k}_{sp}^{^{\prime\prime} }}$ is the damping term of the artificial surface wave respectively.

**Figure 11:**
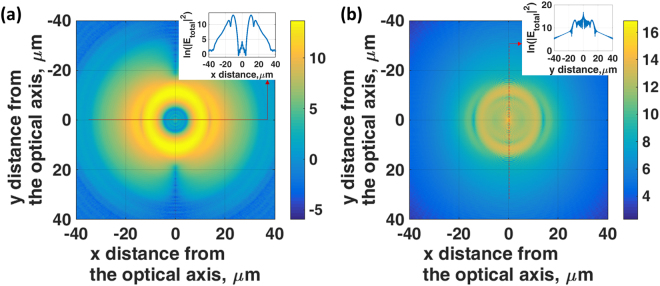
Shows natural log scale of total *|E|*^2^ distribution on a uniform gold surface (46 nm thick with complex permittivity of −9.22 + 1.17i) for (**a**) the conventional SP wave with no artificial phase profile modulation and (**b**) the s-polarized artificial surface wave generated by the SLM phase pattern in Eq. (10) with *(a*, *k*_*p*_*)* = *(19e3*,*0*.*0104)*. The incident light in the calculation was linear polarized with 633 nm wavelength through an NA1.49 objective with coupling oil immersion index of 1.52 at +6 μm defocused distance. Note that the direction of *p-*polarization is along the *x-*axis and the *s-*polarization is along the *y*-axis. The inset on Fig. 11b shows a linescan image of the *|E|*^2^ distribution along the *s-*polarization direction (*y-*axis).

Although we can think of the SLM as simply imposing a phase profile on the returning wave, this has more profound consequences in so much as the phase distribution generates the same field profile on the sample (and/or detection plane) that would be produced with a physical backward propagating surface wave. To demonstrate this, we performed a set of Fresnel equations simulation calculating total intensity distribution on a uniform gold surface (46 nm thick with recovered complex permittivity of −9.22 + 1.17i explained later in the results and discussion section) as shown in Fig. 11 for (a) the conventional SP wave with no artificial phase profile modulation and (b) the s-polarized artificial surface wave generated by the SLM phase pattern in Eq. (10) with *(a*, *k*_*p*_) = *(19e3*, *0*.*0104*). The incident light in the calculation was linear polarized with 633 nm incident wavelength through an objective of numerical aperture 1.49 with coupling oil immersion index of 1.52 at +6 μm defocused distance. For the conventional SP shown in Fig. 11a, there was no wave along the *y-*axis (*s-*polarization direction); the SP wave propagates outwards along the *x-*axis (*p-*polarization direction) and the energy at the center of the optical axis is very low. On the other hand, for the artificial wave case we can see that the SP wave along the *x-*axis is weak and there is a strong surface wave excitation along *y-*axis where this wave focuses down towards the center of the optical axis, as shown most clearly on the inset to Fig. 11b.

These artificial *s-*polarized surface waves do not have ohmic loss, since there is no dip in the modulus of the reflectivity as observed in the BFP. Hence ${k}_{ASP}^{^{\prime\prime} }={k}_{c,ASP}^{^{\prime\prime} }$ and the attenuation equation can then be expressed as:

12 $$ASP(z)=2\gamma {k}_{cz,ASP}^{^{\prime\prime} }{e}^{z{k}_{cz,ASP}^{^{\prime\prime} }}$$

Where *ASP* is the magnitude of artificial surface wave as a function of z defocus and *k*″_*cz*,*ASP*_ is the coupling attenuation of the artificial surface wave.

The linear fit on the ln(ASP(z)) curve allows us to determine *k*″_*cz*,*ASP*_ from the slope and ln(2γk″_*cz*,*ASP*_*)* from the *y*-intercept. Hence the term *γ* can be readily determined allowing us to separate the terms *k*″_*Ωz*_ and *k*″_*cz*_. This artificial surface wave excitation can thus be employed to perform an internal calibration.

## Results

In this section, we demonstrate the accuracy and robustness of the described attenuation measurement method by following each of the steps explained in the Materials and Methods section.

### Total attenuation measurement under the confocal microscope configuration

**Figure 12:**
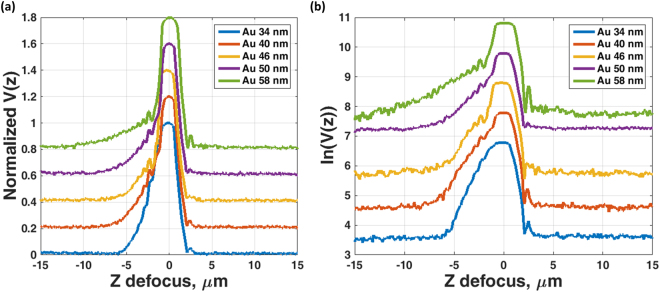
Shows (**a**) Normalized experimental *V(z)* attenuation measurements for 34 nm (blue curve), 40 nm (red curve), 46 nm (yellow curve), 50 nm (purple curve) and 58 nm (green curve) thick of gold samples and (**b**) Natural log scale of the *V(z)* curves. The curves in (a) and (b) have been offset by 0.2 and 1 respectively for a clearer illustration.

The method was verified by preparing plasmonic gold samples onto standard coverslips by sputter coating and the gold thicknesses were confirmed by a surface profiler (model: P10 from KLA Tencor). We then measured the total attenuation *k*″_*sp*_ by the *V(z)* measurements as shown in Fig. 12a. The experimental results in Fig. 12a were then analyzed by taking natural log scale and linear fitting around the z defocus of −5 µm to −3 µm for the Au sample with 34 nm thick and the z defocus of −6 µm to −3 µm for the samples with the 40 nm to 58 nm thick to determine *k*″_*sp*_ as shown in Fig. 12b. Note that the reason for using different z defocus range was that for the 34 nm case the attenuation was very high and the signal vanished before −6 µm defocus as can be observed in Fig. 12a,b. The determined total attenuation coefficients *k*″_*sp*_ are found to be 0.3568 µm^−1^, 0.2391 µm^−1^, 0.1916 µm^−1^, 0.1534 µm^−1^, and 0.0962 µm^−1^ for 34 nm, 40 nm, 46 nm, 50 nm and 58 nm respectively.

These values and the trend agree quite well with the values reported by Kolomenski *et al*.^17^ as shown in Table 1. Each sample was measured 30 times to test the robustness and repeatability of the measurements. The maximum error in recovered attenuations for all the samples were within 1.2%. The errors associated with measurements of each thickness value are shown in Table 1. This indicates that this modified confocal technique can provide a robust attenuation measurement compared to other measurement methods, where the typical value of percentage error is either not fully quantified for SP or for mechanical surface waves the percentage error in the attenuation was 50 times greater than the error in the real part of the *k-*vector^30^.

**Table 1:** Shows the attenuation coefficient *k*″_*sp*_ (in μm^−1^) for confocal *V(z)* measurement and values reported by Kolomenski *et al*.^17^.

Au thicknesses (nm)	*k*″_*sp*_ for confocal *V(z)* measurement		*k*″_*sp*_ extracted from ref.^17^
	Mean value (µ)	Variation coefficient $\sqrt{{\sigma }^{2}}/\mu$	
34	0.3568	0.0029	0.2322
40	0.2391	0.0107	0.1600
46	0.1916	0.0067	0.1123
50	0.1534	0.0084	0.1039
58	0.0962	0.0111	0.0821

There are some discrepancies due to the fact that the surface roughness of the samples may not be the same as the ones used by Kolomenski *et al*.^17^ and the uncertainty (±2 nm) of the surface profiler in sample thickness measurements. Kolomenski *et al*.^17^ reported that the attenuation length (1/*k*″_*sp*_) depends on the roughness of the gold surface and complex refractive index value of gold used in their model; where they used the refractive index value reported in D.E. Gray^31^. These factors could lead to around ±2 μm discrepancies in the attenuation length^17^.

### Confocal surface plasmon microscope as a tool to determine the complex permittivity of gold

We can then take the results in Table 1 and follow the complex *k*_*sp*_ contour calculation protocol described in the transmission line model in the Materials and Methods section.

**Figure 13:**
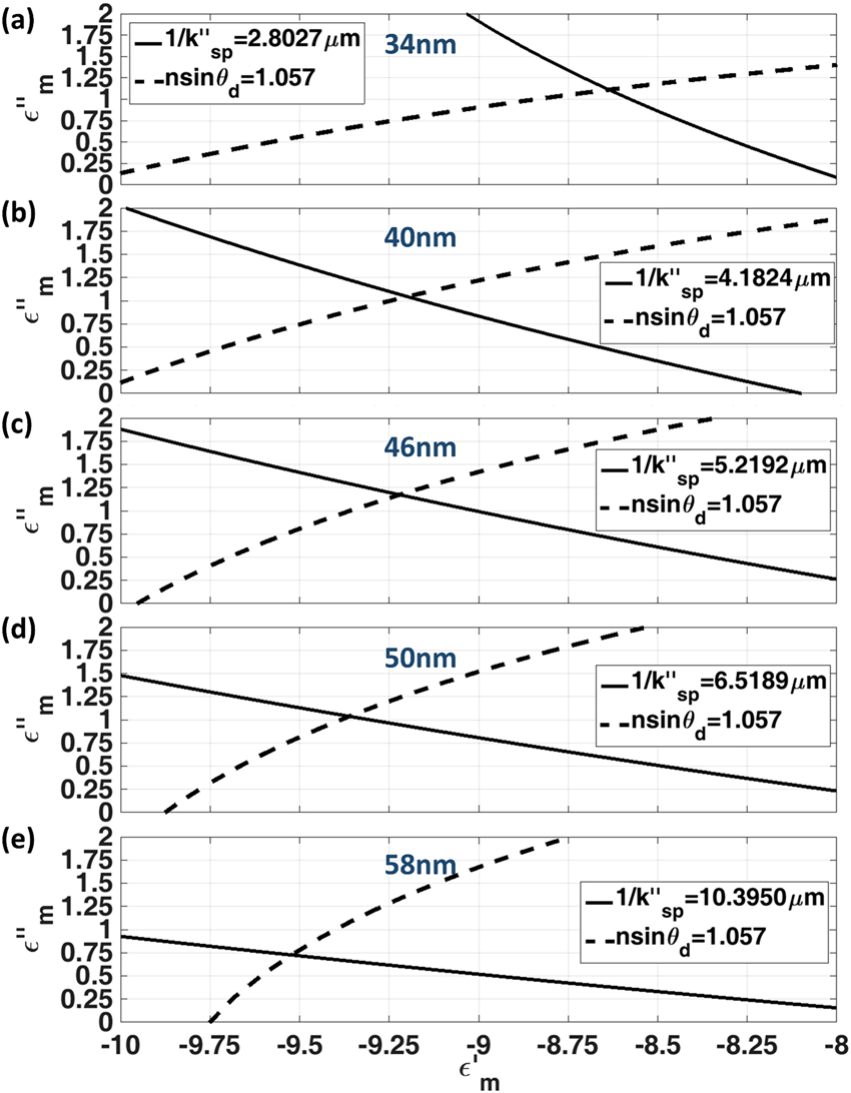
Shows the complex *ε*_*m*_ values that satisfy: the attenuation values as shown in Table 1 shown in black curves and position of the plasmon angle in the BFP of *nsinθ*_*d*_ = 1.0570 shown in dashed-black curves. (**a**) 34 nm, (**b**) 40 nm, (**c**) 46 nm, (**d**) 50 nm and (**e**) 58 nm.

Figure 13 shows the complex *ε*_*m*_ curves that satisfy (a) position of the plasmon angle in the BFP of *nsinθ*_*d*_ = 1.0570 shown in dashed-black curves and (b) the attenuation values as shown in Table 1 shown by the black curves. The intersection between the two conditions (a) and (b) gives the complex permittivity of the gold as listed in Table 2 for all the thicknesses of the gold samples.

**Table 2:** Shows the recovered complex permittivity of the gold samples.

Au thicknesses (nm)	Recovered complex permittivity *ε*_*m*_
34	−8.64 + 1.11i
40	−9.20 + 1.04i
46	−9.22 + 1.17i
50	−9.36 + 1.04i
58	−9.52 + 0.73i

The results in Fig. 13 allows us to work out the complex permittivity of the gold for all the samples from the intersection points as summarized in Table 2. The recovered values except for the 34 nm case were well within the complex permittivity of gold at 633 nm wavelength reported in the literature, for example, −11.7532 + 1.2596i reported by Johnson and Christy^32^, −10.5749 + 1.2765i by Rakić *et al*.^33^, −13.0124 + 1.0331i by McPeak *et al*.^34^, −10.8 + 0.76i by D. E. Gray^31^, −9.10 + 1.00i by E. D. Palik^35^ and −9.60 + 0.62i for gold layer with 2 nm roughness reported by Kolomenski *et al*.^17^. We will show in the later section that the measurement for the 34 nm case was a slight outlier because the relative roughness of thin gold samples is usually found to be greater^36^.

### Separating SP loss mechanisms using Goos-Hänchen phase shift engineering using a phase spatial light modulator

**Figure 14:**
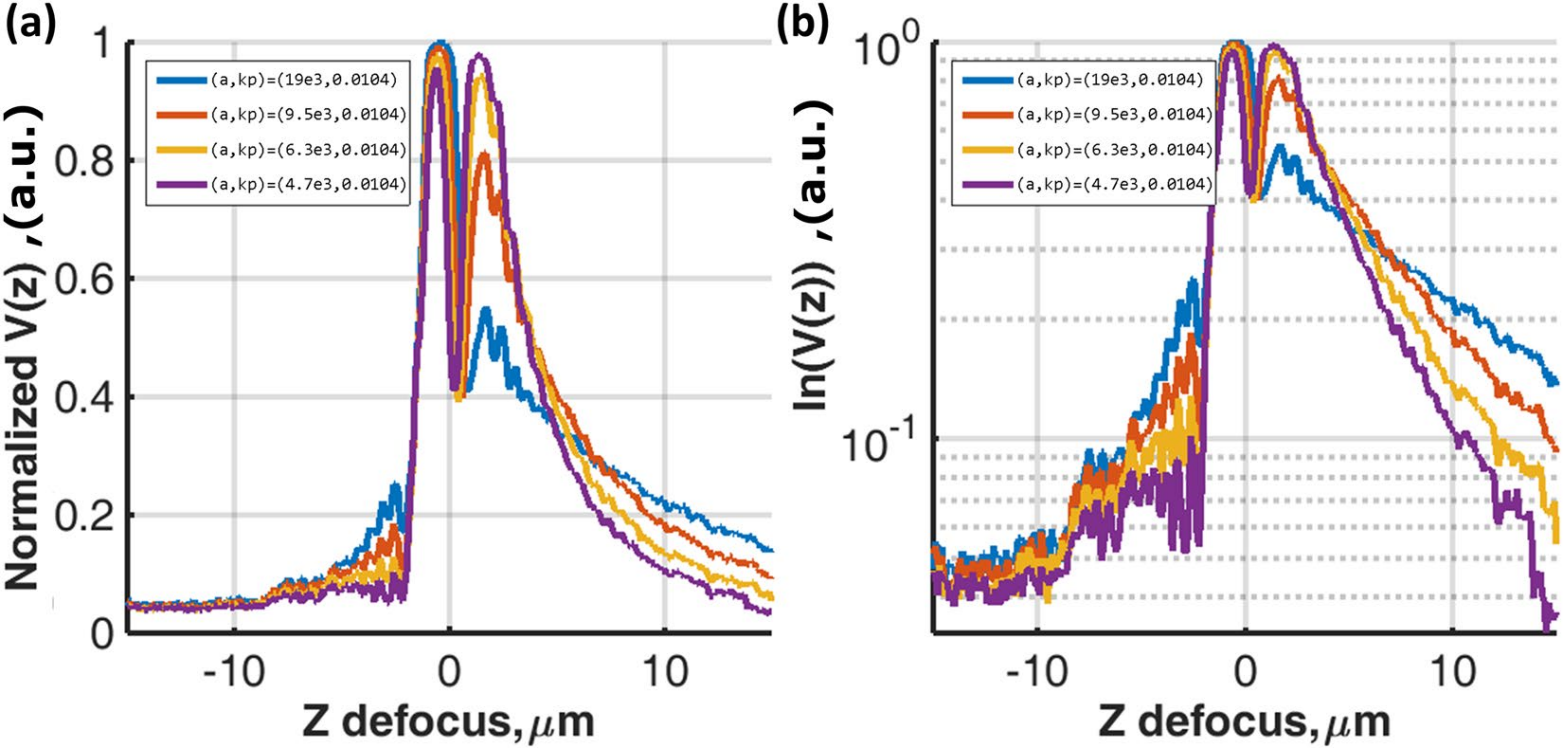
Shows (**a**) Normalized experimental *V(z)* curves for artificial surface wave for the sample with 46 nm thick and (**b**) natural log scale for the results in (a). For *(a*, *k*_*p*_*)* = *(19e3*, *0*.*0104)* for blue curve, *(a*, *k*_*p*_*)* = *(9*.*5e3*, *0*.*0104)* for red curve, *(a*, *k*_*p*_*)* = *(6*.*3e3*, *0*.*0104*) for yellow curve and *(a*, *k*_*p*_*)* = *(4*.*7e3*, *0*.*0104)* for purple curve.

**Figure 15:**
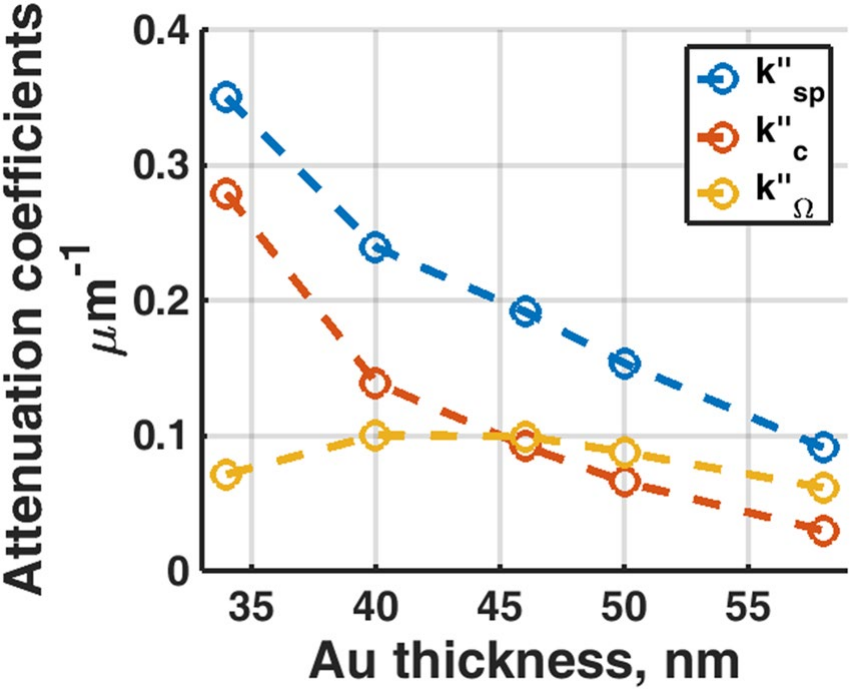
Shows the recovered *k*″_*Ω*_ (red curve), *k*″_*c*_ (yellow curve) and *k*″_*sp*_ (blue curve) for the gold samples with thickness of 34 nm, 40 nm, 46 nm, 50 nm and 58 nm.

Having explained in the Materials and Methods section that we make use of SLM excited s-polarization surface wave to self-calibrate the instrument dependent parameter *γ*, here four sets of artificial phase gradients as shown in Fig. 9 have been employed to ensure the *γ* value determined is reliable, repeatable and independent of propagation length of the artificial surface waves. Figure 14 shows *V(z*) responses corresponding to the 4 artificial phase gradients for 46 nm gold sample. For each sample, the recovered *γ* values were within 5% deviation for all cases. This mean value of *γ* was then inserted into equation 5 to separate the two components of attenuation. It can be seen from Fig. 15 that the crossover point between *k*″_*Ω*_ and *k*″_*c*_ occurs close to 46 nm, where the minimum dip occurs. The simplified Green’s function model predicts the minimum dip at zero intensity level as shown in Fig. 2a for the case *k*″_*c*_/*k*″_*Ω*_ = 1.00; the cross over point between *k*″_*Ω*_ and *k*″_*c*_. This thickness agrees with the optimal thickness that provides the minimum dip in the reflection spectrum for the SP gold sensor^37^. We have also cross checked the complex permittivity of gold recovered, −9.22 + 1.17i (see Table 2), in the earlier section by running a Fresnel model to determine the optimum thickness of gold that gives the minimum dip intensity and found that the minimum dip does occur at 46 nm. This is strong confirmation with the 46 nm recovered from the confocal measurement.

### Validation against the simplified Green’s function

**Figure 16:**
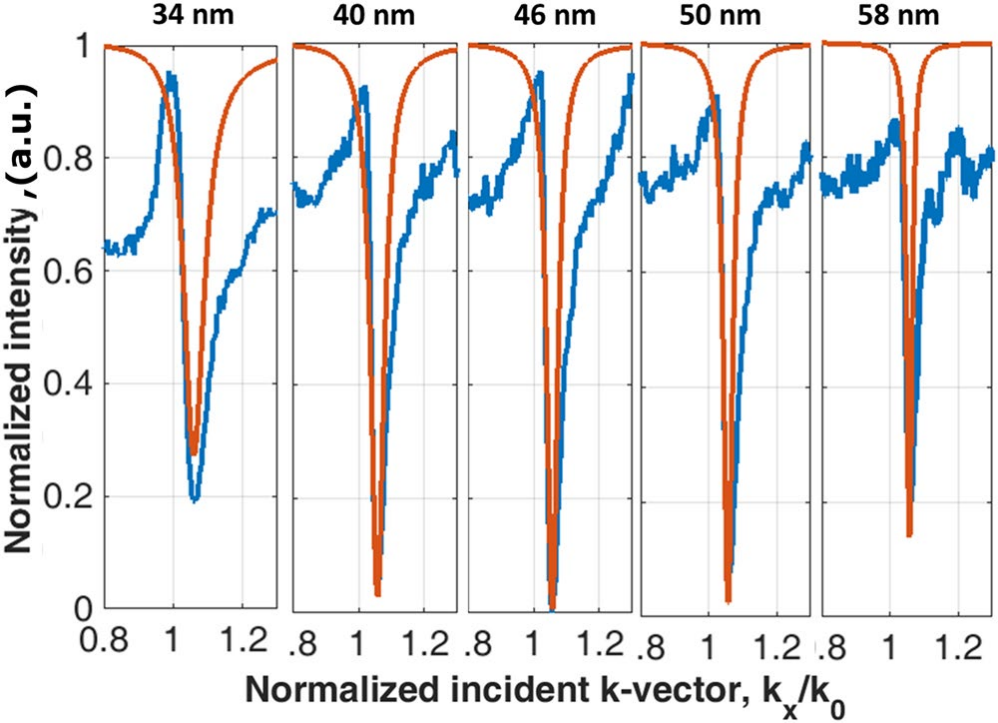
Shows the measured SP dips (blue curves) taken by the BFP camera in comparison with the calculated SP dips (red curves) calculated by the recovered *k*″_*Ω*_ and *k*″_*c*_ in Fig. 15 for all the Au samples.

The recovered attenuation coefficients in Fig. 15 were then validated against the SP dip measurement in BFP by calculating the expected SP dip amplitude distribution by substituting the recovered *k*″_*Ω*_ and *k*″_*c*_ in Fig. 15 into Eq. (1) and Eq. (2) and working out an expected reflection spectrum as shown in Fig. 16. The results except the 34 nm Au show a very good agreement between the two independent measurements indicating that, firstly, the recovered attenuation coefficients were reliable and, secondly, the simplified Green’s function can provide a good approximation to the loss mechanisms of the SP even though the model does not account for the non-symmetric shape of the SP dip in the Kretschmann configuration. The non-symmetric shape of the SP dip is due to symmetric mode (short range SP) for the incident *k-*vector below the plasmonic dip and antisymmetric mode (long range SP) for the incident *k*-vector above the reflectivity minimum^38^. It has been reported that the short-range SP has the higher excitation coefficient compared to the long range SP^39^, where most of the SP excited is the short range mode. We believe that this explains the reason why the simplified Green’s model reflection spectra agree very well with the lower *k-*vector side of the BFP SP dips. The reason that the 34 nm case did not match very well was probably due to the roughness and the thin film quality^36^.

## Discussion and Conclusions

This paper has demonstrated how a modified confocal microscope can be used to measure attenuation of surface waves over a localized region. This can be used to provide measurement of the material properties of the metal layer on a microscopic scale with results that are consistent with those presented in the literature. The main result of the paper, however, is to show how the system can be used to measure attenuation coefficients associated with different loss mechanisms as a direct far-field method and without reference to any model. The measurements were carried out by superimposing an engineered artificial surface wave phase profile with the Goos-Hänchen phase shift opposite to the surface plasmons in the BFP.

Here we take the advantage of the input linear polarization because we can impose a phase profile on the *s-*polarization portion of the beam which mimics the effect of backward propagating surface wave. This allows the *s-*polarization backward surface wave to be artificially excited, where the linear polarization gives a real advantage in this case. In this paper, we have demonstrated that the phase SLM can be employed to replicate the backward surface wave effect with no requirement of such a complicated structure. This backward surface wave enables us to separate the losses and provides an internally self-calibrated measurement. We also show that this method gives accurate results by comparing with independent measurements, such as, back focal plane measurement and mathematical models, such as, a simplified Green’s function and the transmission line model. Although our results have been demonstrated on gold films supporting SPs they are applicable to other surface wave modes involving dielectric layers^40^. Moreover, the measurement concept with some modifications can be applied to characterize chiral structures^41^, films with etched gratings^42^ as well as metamaterial surfaces^43^.

It is important to point out that although all the methods mentioned here in this paper including ours do not distinguish between the ohmic loss and scattering loss where it is still an on-going research^8^, our proposed method does provide a more robust, model-free and a direct measurement of attenuation in far field.

## Electronic supplementary material

SUPPLEMENTARY INFO
