# Supplementary material for: Application of confocal surface wave microscope to self-calibrated attenuation coefficient measurement by Goos-Hänchen phase shift modulation
Source: Sci Rep. 2018 Jun 4;8:8547. doi: 10.1038/s41598-018-26424-2 (PMC5986803; doi:10.1038/s41598-018-26424-2)
Supplement: Supplementary file 1 — SUPPLEMENTARY INFO [file 41598_2018_26424_MOESM1_ESM.docx]

**Supplementary information**

**Application of confocal surface wave microscope to self-calibrated attenuation coefficient measurement by Goos-Hänchen phase shift modulation**

Suejit Pechprasarn^1,2†^, Terry WK Chow^1^ and Michael G. Somekh1,3†[[1]](#footnote-1)↵

*S1 Experiment Description*


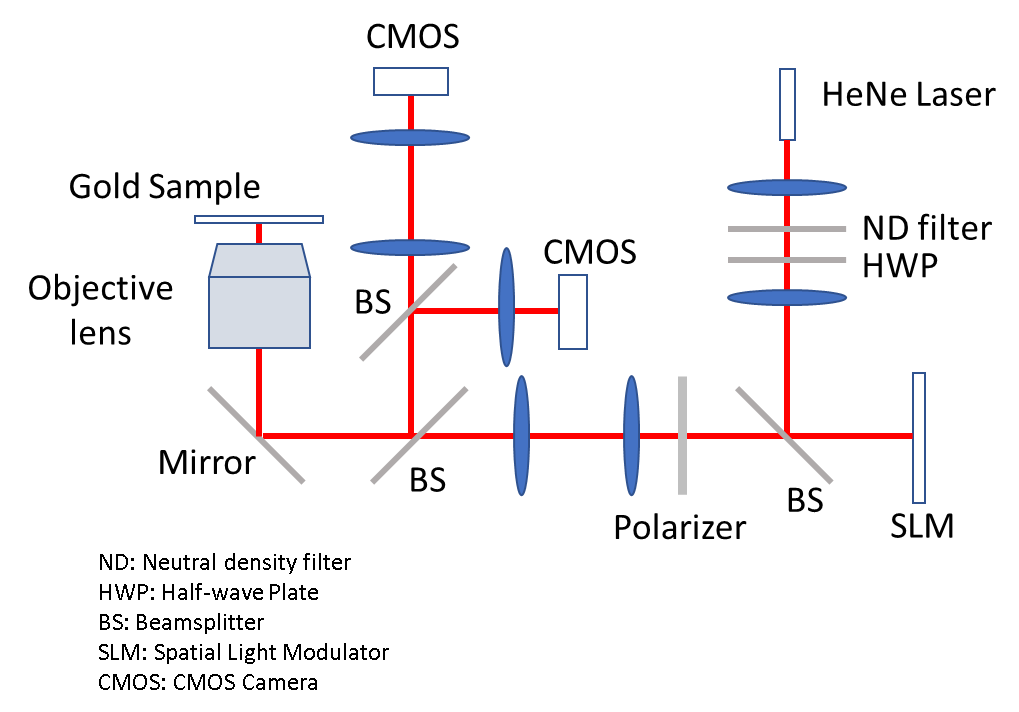


Fig. S1 System diagram of confocal surface plasmon microscope showing all optical components in the system.

The system employs a 1.49NA oil immersion objective lens (Nikon, CFI Apo TIRF 60x H) with oil immersion refractive index (n) 1.52 (Olympus, type F) providing sufficient *k*-vector to excite SP in a high refractive index medium. The phase-SLM (Holoeye LETO, LCOS-reflective, PAN, 1920 x 1080 pixels, 12.5 x 7.1 mm, 256 grey levels, Pixel Pitch 6.4 μm) is aligned on the conjugate plane (BFP) to the objective lens allowing the operations as described in the section “A modified confocal microscope and attenuation measurement” in the main manuscript. The phase-SLM enables (i) defocusing illumination by providing defocused phase pattern in the conjugate plane (BFP) of the objective lens and (ii) an apodization profile (amplitude pupil function) using phase-antiphase pairs pattern on the phase-SLM.

The collimated input beam to the phase-SLM is magnified from a HeNe laser (Melles Griot, 25-LHP-991, Linear polarized, 632.8nm, 10mW). It is worth noting that the half-wave plate (Thorlabs, WPH10M-633, 633nm, zero order) in front of the phase-SLM is to ensure the polarization of the input beam is parallel to the SLM panel where the nematic liquid crystal is aligned parallel otherwise the input beam will not be modulated properly. The modulated beam after phase-SLM is then projected onto the BFP of objective lens and focused onto the gold sample. (The deposition procedure will be described latter). The polarizer between the phase-SLM and the objective lens ensured a pure linearly polarized beam onto the gold sample. The reflected light from the gold sample is collected by the objective lens and projected to the CMOS camera (Thorlabs, DCC3240M, 1280 x 1024 pixels, pixel size 5.3μm). The camera was aligned in the conjugate focal plane of the objective lens to perform virtual confocal pinhole detection. With 2250x magnification, the Airy disk (1.22λ/NA = 0.518μm) can be captured by the camera (corresponding to a field of view on the sample of 3.02μm x 2.41μm). The confocal signal is computed by summing the intensity within the virtual confocal pinhole. An additional CMOS camera (same model) was aligned in the BFP of the objective lens to capture the BFP image as shown on Fig.4. Moreover, achromatic doublets (Thorlabs, AC Coated, 400-700nm) and pellicle beamsplitters (Thorlabs, CM1-BP145B1) were used in the system to minimize aberration and the phase distortion.

*S2 Sample Preparation*

The gold layer was sputtered at 70W on coverslip (No. 1) under deposition pressure of 10^-2^ torr with nominal deposition rate of ~2nm/s. The thicknesses of the gold samples were then measured by Surface Profiler (Tencor P-10).

*S3 Basis of simplified reflection coefficient*

The simplified reflection coefficient is obtained assuming an appropriate Green function for a line excitation as explained in Somekh 2007^1^. Each line source is assumed to generate two surface wave contributions one propagating from left to right and the other propagating from right to left. The propagation vector of each contribution is *k_p_* corresponding the complex wave number of the surface plasmon.

For a unit incident plane wave with the wavenumber *k_x_*, the field *E_ref_*  at an arbitrary position *x_0_* is obtained by adding the contributions propagating from left to right and right to left respectively.

$E_{sp}\left(k_{x},x_{0}\right)=2k_{c}^{''}\left[\int_{-\infty}^{x_{0}} \exp ik_{x}x\exp ik_{p}\left(x_{0}-x\right)dx+\int_{x_{0}}^{\infty} \exp ik_{x}x\exp-ik_{p}\left(x_{0}-x\right)dx\right]$ (S1)

where the $k_{c}^{''}$is the coupling coefficient for the surface plasmons which arises from the pole contribution of the reflection coefficient as explained in the analogous case of surface acoustic waves in Bertoni 1973^2^. Integrating out this contribution gives the expression for the incident field $\exp ik_{x}x_{0}$ multiplied by the contribution reflection coefficient due to surface plasmon excitation given in equations (1) and (2).

Adding this expression and to the direct reflection of -1 gives the approximate form of the reflection coefficient given in equation (1). In the present work, it is important to eliminate the direct reflection to retain the contribution of the surface wave, which in the confocal microscope is done by defocusing.

*S4 V(z) derivation*


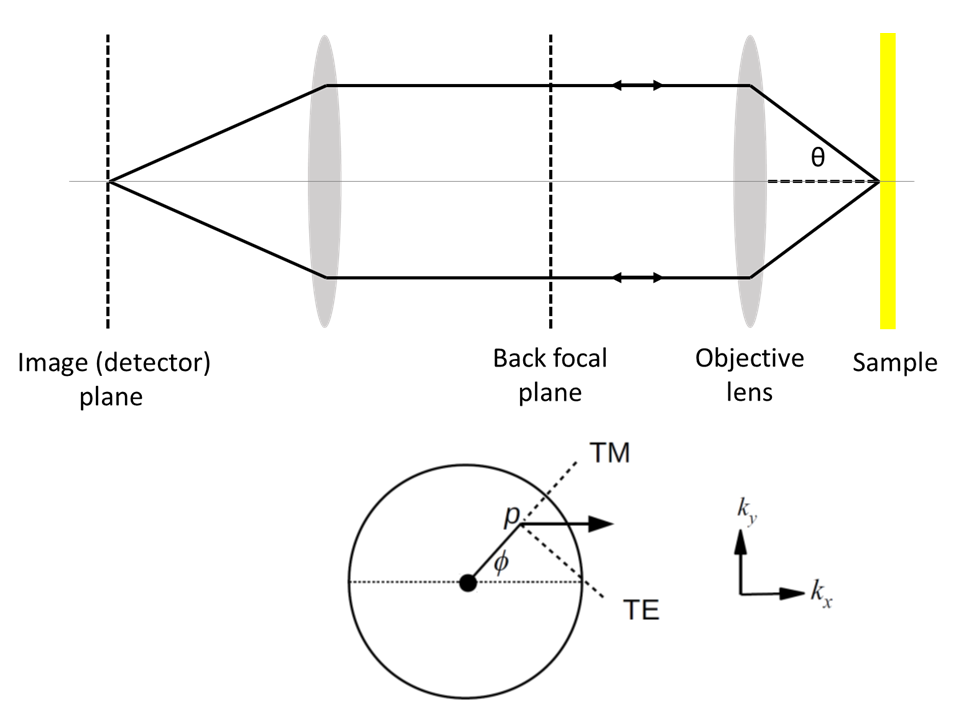


Fig. S2 Schematic diagram showing derivation of V(z). Upper figure shows light path (omitting the coupling optics for clarity). The light from the sample is imaged onto the image (detector) plane where a pinhole us used to select light emerging from the focus. A spatial light modulator (not shown) controls the amplitude and phase on the back focal plane. The lower figure shows the linearly polarized incident light resolved into transverse magnetic (TM) and transverse electric (TE) components.

The idea of *V(z)* has been widely used in acoustic microscopy where the acoustic transducer is phase sensitive. The idea was extended to surface plasmons in ^3^ where the system employed was a heterodyne interferometer. We will now derive the expression for *V(z)* with reference to figure S2 and pay special attention to the role of the pupil function and the reflection coefficient.

We consider a linear polarized light input field into the back focal plane. Other polarization states, such as circular and radial, are used but critical in our measurement in this paper is the use of a polarization that gives azimuthal variation. All the positions in the back focal plane map linearly to spatial frequencies on the image plane, so for simplicity we will simply equate positions in the back focal plane to *k_x_* and *k_y_*. The SLM is placed conjugate to the back focal plane; this controls the amplitude and phase of the spectrum passing onto the sample. We can represent the combined effect of the SLM and the objective lens as *P_1_(k_x_,k_y_).* Incident light oriented tangentially in the back focal plane will generate *s*-polarized light (TE) and radially oriented light will produce *p-*polarized (TM) light on the sample, it is the latter polarization, of course, that generated the SPs.

Let us now examine the light reflected from the sample at an arbitrary position in the back focal plane as shown in the lower part of figure S2. The light reflected from sample in focus to the position ‘p’ in the back focal is given by:

$P_{1}(k_{r},\varphi)\left(r_{p}\cos^{2}\varphi+r_{s}\sin^{2}\varphi\right)$ (S2)

Where we have rewritten the pupil function in polar coordinates so that

$\text{k}_{\text{x}}=\text{k}_{\text{r}}\cos\varphi$ and $\text{k}_{\text{y}}=\text{k}_{\text{r}}\sin\varphi$where $\text{k}_{\text{r}}=\sqrt{\text{k}_{\text{x}}^{2}+\text{k}_{\text{y}}^{2}}$, with the wavenumber of the incident wave given by: $\text{k}=\frac{2\pi n_{coup}}{\lambda}$, where *λ* is the free space wavelength and *n_coup_* is the refractive index of the couplant of the immersion objective. Note also $k_{r}=k\sin\theta$, where *θ* is the angle of incidence.

This contribution to the signal can be readily seen by noting that the *x-*polarized beam is resolved along the radial and tangential directions to be effected by the reflection coefficients for *p-* and *s*- respectively.

Since *r_p_* and *r_s_* are not equal there is also a *y*-polarized component of the field. Although this can be quite significant particularly at large angles of incidence the symmetry of the confocal arrangement means that all these contributions cancel at the center of the detection plane (the confocal pinhole) so we will ignore this component.

The field given by eq. (S2) is subject to application of the pupil function on return, denoted as *P_2_*, if the light interacts with the SLM a second time *P_2_* is generally equal to *P_1_*. In our system, the light interacts with the SLM on incidence but not on reflection so *P_1_* and *P_2_* are not equal, notwithstanding this, for a planar sample the two contributions can be combined into a single pupil *P* which is the product of each pupil. In general, and particularly in our experiments, the sample is not at focus, so we need to add a phase factor to account for these effects. Taking these factors into consideration we obtain a general expression for the *x*-component of the returning field, $E_{bfp}(k_{r},\varphi)$at a particular defocus *z.*

$E_{bfp}(k_{r},\varphi)=P\left(k_{r},\varphi\right)\left(r_{p}(\theta)\cos^{2}\varphi+r_{s}(\theta)\sin^{2}\varphi\right)\exp(2ik\cos\theta z)$ (S3)

In order to find the field at the pinhole we note that since the field in the back focal plane is a Fourier transform of the field at the image plane the field is just proportional the integral of the whole field. This expression is only exactly correct for an infinitely small pinhole which is, clearly, impractical. In practice, a pinhole diameter about ¼ the size the Airy disc gives a very close approximation to an ideal confocal response^4^ and acceptable signal to noise ratio. We can therefore write the measured output signal $\left|V(z)\right|^{2}$as:

$\left|V(z)\right|^{2}=\left|\iint_{aperture} P\left(k_{r},\varphi\right)\left(r_{p}(\theta)\cos^{2}\varphi+r_{s}(\theta)\sin^{2}\varphi\right)\exp(2ik\cos\theta z)\text{ }k_{r}\text{ dk}_{\text{r}}d\varphi\right|^{2}$ (S4)

We can now see the different terms interact. For a simple pupil function with no phase variation the rate of change for negative defocus changes slowly at two positions, close to normal incidence the phase term in the exponent changes slowly with angle and at the position close to the excitation of the surface wave where the phase shift of the reflection coefficient is cancelled by the phase shift due to defocus as shown in figure 7. In our present experiments, however, we do not want interference with the light at normal incidence so the pupil function is set to zero for low angles of incidence. The major contribution to the output is thus due to the region where the total phase shift due to defocus and the reflection coefficient changes slowly.

Eq. (S4) tells us that as far as the *V(z)* response is concerned the phase distribution may be imposed with the pupil function, the reflection coefficient or defocus or a combination of these. For instance, we may get the effect of defocus by remaining in focus and imposing the appropriate phase shift onto the pupil function^5,6^. By the same argument we may generate a similar reflected light distribution on a surface that does not excite surface waves by using the SLM to generate the phase distribution corresponding to SP excitation. This is indeed what is done in the present paper where a phase distribution corresponding to a negative Goos-Hänchen shift is imposed with the SLM. We thus use the pupil function to generate the same distribution that would be produced by a sample with the negative Goos-Hänchen shift.

It is worth making some further observations on the form of the pupil function. We mentioned that it should block the light coming from low angles of incidence. As discussed in the Simplified Green function in the main text when light is close to the optimum angle for excitation of SPs some the light is converted to SPs and some is directly reflected. For our measurement, it is necessary that the light emitted directly is blocked by the pinhole so that the microscope detects only the light arising from excitation of surface waves, as shown in Fig. 6. In terms of the simplified pupil function this means blocking the ‘-1’ term and retaining only the surface plasmon expression. To get an order of magnitude estimate we make the following considerations. Consider the passband of a rectangular pupil function of width *k_min_* to *k_max_*, of course, the pupil is Gaussian but the following simplifications suffice to give an intuitive picture. To equate the Gaussian pupil with the rectangular one we define *k_min_* and *k_max_* to give exactly the same standard deviation as the Gaussian pupil shown in Fig. 4. We need to defocus sufficiently so that the directly reflected rays miss the pinhole or equivalently there is strong phase cancellation of this reflection across the back focal plane.

This may be expressed as: $\left|z\right|\left(\sqrt{k^{2}-k_{\min}^{2}}-\sqrt{k^{2}-k_{\max}^{2}}>\pi\right)$ . This states that the phase variation across the direct reflection is >*2π*. Equating our Gaussian pupil to the rectangular pupil gives *k_min_*=0.61*k* and *k_max_*=0.78*k*, for an annular pupil extending over this range the condition for *z* is met for defocus beyond approximately 1.3 microns, so the simulations which show little effect of the direct reflection beyond 3 microns are borne out.

*S5 A simple analysis of the GH shift with a defocused high NA beam.*

The GH shift for the focused beam takes a very different form from the effect with a paraxial beam. The analysis presented in the paper uses the reflection coefficient from the Fresnel equations and a pupil function corresponding to spherical lens with input linear polarization. In this section for the purpose of illustration, we show how a beam with large angular range will generate a decaying wave when defocused.

We consider a cylindrical lens with a large aperture (Fig. S3), this will illustrate the key points while keeping the algebra as simple as possible. To get an analytical expression we will apply the principle of stationary phase^5,6^ twice, firstly to get an asymptotic representation of the field on the sample surface and, secondly, to calculate the field distribution due to the surface plasmons on the surface. Let us initially use the stationary phase approximation to derive the field distribution on the sample when the sample is defocused by a ‘sufficient’ distance.

We consider the lens has a pupil function *P(s),* where *s* is the sine of the incident angle, *θ*.

We can write the field on the sample as a weighted sum of all the plane wave components as function of defocus. We thus have

$E(x,z)=\int_{s_{\min}}^{s_{\max}} P(s)\exp ik\left(\cos\theta z+sx\right)ds=\int_{s_{\min}}^{s_{\max}} P(s)\exp ikz\left(\sqrt{1-s^{2}}+s\beta\right)ds$ (S5)

Where the limits of integration are the minimum and maximum sines of the incident angle and *k* is defined in section S4. In the second equality, we have replaced the *x* with *β*=*x/z*. As this renders the equations much simpler and has clear physical meaning when the field distribution is interpreted in ray-optical terms.


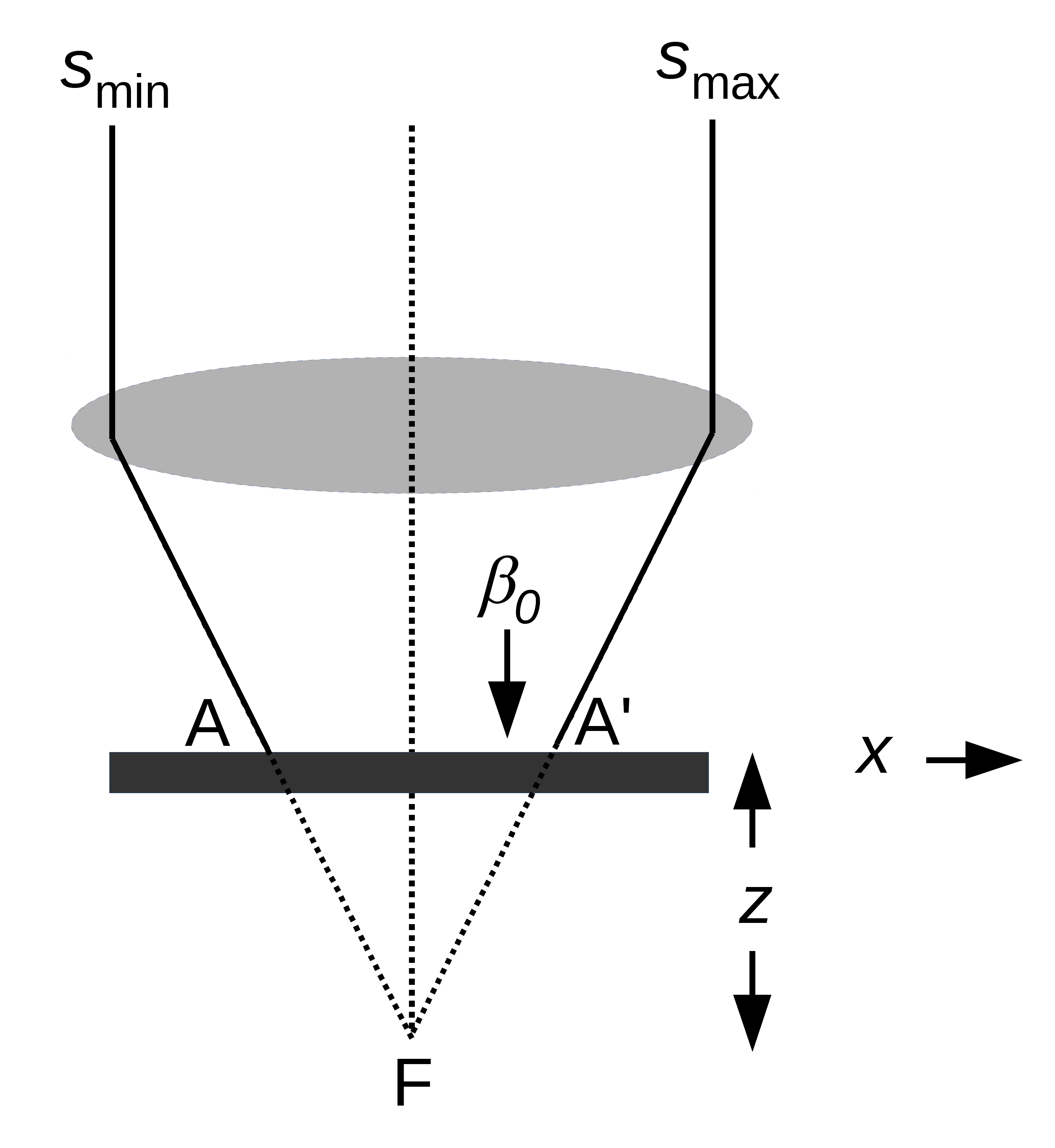


Fig. S3 shows the path length between the focus and the point of intersection with the sample

The phase term, *φ*, in the integral is zero when: $\beta=\frac{s}{\sqrt{1-s^{2}}}$, at this point the second derivative is given by: $\frac{d^{2}\varphi}{ds^{2}}=-\frac{kz}{\left(1-s^{2}\right)^{3/2}}=-kz\left(1+\beta^{2}\right)^{3/2}$ (S6)

The asymptotic stationary phase solution is this given by:

$E\left(\beta,z\right)\approx P(\beta)\sqrt{\frac{2\pi}{kz}}\left(1+\beta\right)^{3/4}\exp i\frac{\pi}{4}\mathrm{sgn}(z)\exp i\varphi\left(\beta\right)$ (S7)

Where *φ(β) *is $-kz\sqrt{1+\beta^{2}}=-\frac{kz}{\cos\theta}$, this is precisely the phase one would calculate from ray optical considerations by taking into account the path length between the focus and the point of intersection with the sample (FA) of figure S3.

The next stage is use this expression to get an estimate of the field distribution arising from the surface wave excitation so each point on the surface will generated a surface wave whose value is determined by the weighted sum of these contributions. The approach here is essentially the same as that applied in section S3 but here the input field is due to a defocused wide angle beam rather than a plane wave.

The field due to the SPs travelling from left to right is given by:

$2k_{c}^{''}\int_{-\infty}^{x_{0}} E(\frac{x}{z},z)\exp ik_{p}(x_{0}-x)\text{ }dx=2k_{c}^{''}z\int_{-\infty}^{\beta_{0}} zE(\beta,z)\exp ik_{p}(\beta_{0}-\beta)\text{ }d\beta$ (S8)

The introduction of the *z* in the last expression arises from$\frac{d\beta}{dx}=\frac{1}{z}$. This term is crucial since in cancels the reciprocal of root *z* terms arising from the stationary phase approximation.

We combine all the constant terms in equation (S7) as:

$E(\beta,z)=A(\beta,z)\exp i\varphi(\beta)$ (S9)

The excited field can therefore be written as:

$2k_{c}^{''}\exp ik_{p}^{'}\beta_{0}z\int_{-\infty}^{\beta_{0}} A\left(\beta,z\right)\exp-(\beta_{0}-\beta)k_{p}^{''}z\exp-ikz\left(\sqrt{1+\beta^{2}}+\beta\sin\theta_{p}\right)d\beta$ (S10)

The stationary phase point for this integral is when:

$\frac{\beta}{\sqrt{1+\beta^{2}}}=-\sin\theta_{p}$, which gives$\beta=-\tan\theta_{p}$, which is the point predicted from the ray optical approximation. The second differential of the phase variation at the stationary phase point is:$\frac{-kz}{\left(1+\beta^{2}\right)^{3/2}}$.

This leads to an expression for the field due to the surface plasmon travelling from left to right at negative *z* as:

$2k_{c}^{''}\sqrt{\frac{2\pi z}{k}}A\left(-\tan\theta_{p},z\right)\left(1+\tan^{2}\theta_{p}\right)^{3/4}\exp\left(ik_{p}\sin\theta_{p}x_{0}+\frac{\pi}{4}\right)\exp-ikz\cos\theta_{p}\exp-k_{p}^{''}(x_{0}+z\tan\theta_{p})$ (S11)

This expression is essentially the expression obtained from a ray analysis and shows that ray interpretation is the stationary phase limit of the wave field. Evaluation of the amplitude terms shows the non-exponential terms in *z* cancel*,* so that the last term in (eq. S11) shows how the GH effect manifests itself with a pure exponentially decaying field as demonstrated by the exact Fresnel analysis.

Note that this term ignores the direct reflection which, in section S4, we have explained is not detected at large defocus.

It should be noted that analysis above shows how the GH shift from a defocused beam leads to exponentially decaying wave. Moreover, the confocal arrangement means that the region of stationary phase operates for both generation of the wavefield and detection, so that the point of detection is complementary to the excitation point given by: $\beta=\tan\theta_{p}$. The analysis above shows that in the limit of the stationary phase approximation the effect of the GH shift on the focused beam is to generate an exponentially decaying tail arising from the surface wave. The confocal pinhole blocks the other components. The region of validity of the approximation is borne out by the calculation from application of the exact Fresnel equation as shown in Fig. 6 in the main text.

*References*

1 Somekh, M. G. in *Optical Imaging and Microscopy: Techniques and Advanced Systems* 347-399 (Springer Berlin Heidelberg, 2007).

2 Bertoni, H. & Tamir, T. Unified theory of Rayleigh-angle phenomena for acoustic beams at liquid-solid interfaces. *Applied Physics A: Materials Science & Processing* **2**, 157-172 (1973).

3 Somekh, M. G., Liu, S. G., Velinov, T. S. & See, C. W. High-resolution scanning surface-plasmon microscopy. *Applied Optics* **39**, 6279-6287 (2000).

4 Zhang, B., Pechprasarn, S., Zhang, J. & Somekh, M. G. Confocal surface plasmon microscopy with pupil function engineering. *Optics Express* **20**, 7388-7397 (2012).

5 Papoulis, A. *The Fourier Integral and its Applications*. (McGraw-Hill, 1962).

6 Cohn,S. Integral Asymptotics 3: Stationary Phase [Online Lecture Note]. Retrieved from https://www.math.unl.edu/~scohn1/8423/intasym4.pdf

1. ^1^Dept of Electronic and Information Engineering, The Hong Kong Polytechnic University, Hung Hom, Hong Kong SAR, China

   ^2^Faculty of Biomedical Engineering, Rangsit University, Pathum Thani, 12000, Thailand

   ^3^Present address: Nanophotonics Research Center, Shenzhen University, Shenzhen, China

   †Correspondence: Suejit Pechprasarn, E-mail: [suejit.pechprasarn@polyu.edu.hk](mailto:suejit.pechprasarn@polyu.edu.hk)

   Michael G. Somekh, E-mail: [mike.somekh@polyu.edu.hk](mailto:mike.somekh@polyu.edu.hk) [↑](#footnote-ref-1)
